# Epidemiology of tendon and ligament injuries in Aotearoa/New Zealand between 2010 and 2016

**DOI:** 10.1186/s40621-020-0231-x

**Published:** 2020-02-10

**Authors:** Sita T. Clark, Mark Zhu, Greg D. Gamble, Dorit Naot, Sarah-Jane Paine, Nicola Dalbeth, Jillian Cornish, David S. Musson

**Affiliations:** 1 Department of Medicine, University of Auckland, Auckland, New Zealand; 2 Department of Orthopaedics, Auckland District Health Board, Auckland, New Zealand; 3 Te Kupenga Hauora Māori, University of Auckland, Auckland, New Zealand; 4 Department of Rheumatology, Auckland District Health Board, Auckland, New Zealand

**Keywords:** Tendon, Ligament, Injuries, Epidemiology

## Abstract

**Background:**

Injuries to tendons and ligaments make up a large portion of musculoskeletal injuries, and contribute to significant morbidity and healthcare costs. However, there is currently a poor understanding of the burden of these injuries at a population level.

The purpose of this study was to quantify the burden and distribution of tendon and ligament injuries in the Aotearoa/New Zealand population.

**Methods:**

Using the Accident Compensation Corporation (ACC, a no fault comprehensive compensation scheme encompassing all of Aotearoa/New Zealand; population in 2013 4.4 million) database, data specific to tendon and ligament injuries were identified between July 2010 and June 2016. The total number of claims made and the total cost of these claims per financial year were analyzed. Injuries were categorized by anatomical site, gender, ethnicity and age of the claimant.

**Results:**

During the 6-year study period, the total number of tendon and ligament injury claims was 1,112,077, with a total cost of over $1.4 billion NZD. There was a 16.2% increase in the number of claims, and a 40% increase in the total cost of these injuries during this period.

The majority of claims were made by people of European ethnicity, whilst the number of claims made by people of Asian ethnicity increased at the greatest rate; 52% (from 9047 claims in 2011) during the 6-year study period. Interestingly, Māori (Indigenous New Zealanders) maintained the highest average cost per claim ($1614.05 NZD); 13% more than the overall average cost per claim ($1262.12 NZD). The most common sites of injury were the shoulder and knee; these injuries were also the greatest contributors to overall cost. The total costs of injuries peaked in claimants aged 40–54, irrespective of the number of claims made for that age group.

**Conclusions:**

Health and economic burdens of tendon and ligament injuries in Aotearoa/New Zealand are rising. The high healthcare costs underscore the urgent need for multifaceted interventions to reduce the incidence and improve clinical outcomes of tendon and ligament injuries.

## Introduction

Diseases and injuries of tendons and ligaments are some of the most commonly diagnosed musculoskeletal problems clinically (Clayton & Court-Brown, 2008; James et al., 2008). In the United States, 33 million musculoskeletal injuries are reported each year, with nearly half of these involving tendons and ligaments (James et al., 2008). Although the majority of such injuries are non-fatal, they can be severely debilitating, resulting in significant reductions in patient quality of life, loss of productivity, and considerable costs to the healthcare system (Liu et al., 2008; Mock & Cherian, 2008; Riley, 2008; Vitale et al., 2007). In the United Kingdom, absenteeism due to lateral epicondylitis (tennis elbow) alone, is approximated to cost £27 million per annum (Walker-Bone et al., 2012). Furthermore, tendons and ligaments heal poorly frequently resulting in months of disability and weaker tendon(s) that are more susceptible to future injuries (Lipman et al., 2018; Riley, 2008). Despite our increased understanding of tendon biology and tendon repair mechanisms, we are yet to develop effective treatment strategies, and surgical outcomes are often poor (Coghlan et al., 2008; James et al., 2008; Kukkonen et al., 2014; Liu et al., 2008; Vitale et al., 2007).

The first step in a public health approach to injury prevention and treatment, is to define the magnitude of the problem and identify the epidemiological characteristics of the issue (Krug et al., 2000). To date, a number of studies have been undertaken in other countries to determine the incidence of specific tendon injuries, such as rotator cuff tears, Achilles ruptures and tears of the hip abductor (Albers et al., 2016; Colvin et al., 2012; De Boer et al., 2014; de Jong et al., 2014; Gianotti et al., 2009; Nyyssonen et al., 2008; Paloneva et al., 2015; Sanders Jr. et al., 2015; Zbrojkiewicz et al., 2018). From these, it is clear that the prevalence of specific tendon/ligament injuries is rising, as is the cost of treating musculoskeletal disorders as a whole, with an aggregate total expenditure on musculoskeletal conditions increasing from US$367.1 billion to $796.3 billion, from 1996 to 1998 to 2009–2011 (Lipman et al., 2018). However, little work has been done to understand the burden of these injuries at the population-level, and no current data exists for Aotearoa/New Zealand (Hopkins et al., 2016). Here, we have undertaken an epidemiological study of tendon/ligament injuries to determine whether this global trend holds true in Aotearoa/New Zealand, and to identify epidemiological trends that can be used to build an informed healthcare approach to tackle this clinical problem at a national level.

We have used data sourced from the Aotearoa/New Zealand Accident Compensation Corporation System (ACC),. Established in 1974, the ACC is a NZ government taxpayer-funded scheme which offers no fault compensation to every individual who suffers an accidental injury in Aotearoa/New Zealand (population according to the last Census in 2013 was 4,242,048), including overseas visitors. People who suffer an injury make a claim to ACC when seeking treatment from a medical professional. When making a claim, information is self-reported by the claimant using standard forms, with key demographic information recorded, including gender, self-reported ethnicity and age. The registered healthcare provider completes the form providing initial diagnosis and the claim is then filed with ACC. Once ACC has accepted a claim, compensation covers costs to the healthcare provider, medical treatment costs, surgery, income replacement (80% of salary), rehabilitation (including work, home and vehicle modifications) and support services.

Being a no-fault compensation scheme, reporting of injuries is likely to be more comprehensive than in most other countries as there is no deterrent for making a claim. This places New Zealand in a unique position to provide detailed descriptive epidemiological data, including costs associated with treatment, which can be used for analysis.

Here, we have utilised this data set to determine the national burden of tendon and ligament injuries within Aotearoa/New Zealand, over a six-year period, and used this descriptive data to identify epidemiological trends.

## Methods

This was a retrospective population-based study, investigating the nationwide epidemiology and burden of tendon and ligament injuries within Aotearoa/New Zealand, during a 6-year period from July 1st, 2010 through to June 30th, 2016.

Anonymized national claims data on tendon and ligament injuries were sourced from, and prepared by, the ACC. When a claim is lodged by a patient, a registered healthcare professional makes an initial diagnosis and assigns a Read code that most accurately matches the patient’s injury. There are over 33,000 Read codes to choose from, so to isolate data specific to tendon and ligament injuries the search terms “Tendon, Tendonitis, Tenosynovitis, Ligament, Epicondylitis and Rotator Cuff” were used to identify relevant Read codes. This produced information from 511 Read codes, which were considered relevant to this study (Additional file 1). We obtained data on the number of claims made for these injuries per year from July 1st to June 30th (total Claim Count) and the costs that these claims accrued over time Cost Ex GST in New Zealand Dollars (NZD)).

Injuries were categorised based on anatomical site of the injury (Shoulder, Elbow, Knee, Ankle, Finger, Thumb & Wrist, Upper & Lower Arm, and Hip, Upper Leg & Thigh). These were then divided into subgroups based on gender (Male and Female), ethnicity (classified as either Māori (the indigenous people), Pacific Peoples, Asian, “Other” ethnicity, and European, using the prioritisation system developed for the NZ health and disability sector (MinistryofHealth, 2017)), and age band at lodgement of claim (0–14 years, 15–19 years, 20–24 years, 25–29 years, 30–34 years, 35–39 years, 40–44 years, 45–49 years, 50–54 years, 55–59 years, 60–64 years, 65–69 years, 70–74 years, 75–79 years, 80–84 years, 85 years and over).

The total number and total cost of claims were produced by ethnicity, age-group and gender. Age-ethnicity specific population estimates from the 2013 NZ Census, sourced from Statistics NZ, served as denominators for the calculation of the age-specific rates for each ethnic group in that year. Rates (with 95% confidence intervals) are reported per 100,000 people.

Specific cost per claim values were calculated (Cost excluding GST divided by the number of claims (Claim Count)). Predicted values for the year 2030 were extrapolated from 6 year data (2011 to 2016) using ordinary least squares (OLS) regression, performed using Prism (Prism version 8.0.0 for Windows, GraphPad Software, San Diego, California USA, www.graphpad.com). All costs are shown in New Zealand Dollars.

## Results

### The burden of tendon/ligament injuries is increasing.

During the study period, the total number of tendon and ligament injury claims accepted by ACC was 1,112,077, and the cost of these claims totaled over $1.4 billion NZD. Annually, the number of claims increased from 170,874 in 2011, to 198,580 in 2016 (a total increase of 16%). There was also a rise in the total annual cost ex GST for the claims, from $202,526,476 to $283,334,932 (a total increase of 40%), and cost per claim, from $1185.24 to $1426.80 (a total increase of 20%) between 2011 to 2016 (Table 1).

**Table 1:** Annual total claim count, total cost and cost per claim for each year within the 6-year study period. All costs are shown in New Zealand Dollars

End of reporting year	2011	2012	2013	2014	2015	2016	6 Year Total
Total Claim Count	170,874	178,902	183,385	184,863	195,473	198,580	1,112,077
Total Cost Ex GST	$202,526,476	$203,975,934	$215,716,748	$233,429,167	$264,591,232	$283,334,932	$1,403,574,489
Average Cost per Claim	$1,185.24	$1,140.15	$1,176.31	$1,262.71	$1,353.59	$1,426.80	$1,262.12

**Fig. 1:**
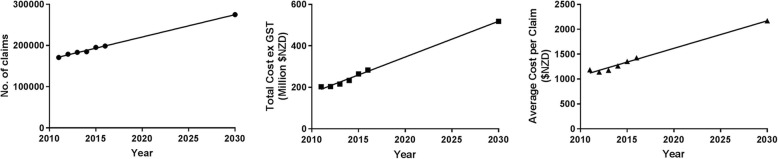
Future predicted trends in tendon and ligament injuries using study data after performing OLS regression. **a** Claim count **b** Cost Ex GST and **c** Average cost per claim

Using the data collected in this study, OLS regression was performed to predict the future burden of tendon injuries. If a linear increase in these claims were to occur, by the year 2030 the total number of claims per year will increase to 274,786; a 38% increase from 2016. Furthermore, the total annual cost ex GST will rise to approximately $518,500,000 (an 83% increase from 2016), and the average cost per claim will increase 52% to $2169.48 (Fig. 1).

### There is a gender disparity in the number and cost of claims made for tendon/ligament injuries

Over the 6-year period reviewed here, there were consistently more claims made by males than females, with males accounting for 56% of all claims made for tendon and ligament injuries (Table 2). According to the 2013 Aotearoa/New Zealand Census, females and males comprised 51.3 and 48.7% of the Aotearoa/New Zealand population, respectively (Additional file 1), suggesting an over-representation of claims made by males.

**Table 2:** Annual total claim count, total cost ex GST and cost per claim per gender for each year, within the 6 year study period

End of reporting year	2011	2012	2013	2014	2015	2016	6 Year Total
Total Claim Count							
Females	73,924	77,618	80,214	81,712	87,002	89,569	490,039
Males	96,950	101,284	103,171	103,151	108,471	109,011	622,038
Total Cost							
Females	$61,342,029	$62,496,249	$67,973,231	$73,495,812	$83,134,428	$88,599,765	$437,041,514
Males	$141,184,447	$141,479,685	$147,743,517	$159,933,355	$181,456,804	$194,735,167	$966,532,975
Average Cost per Claim							
Females	$829.80	$805.18	$847.40	$899.45	$955.55	$989.18	$887.76
Males	$1,456.26	$1,396.86	$1,432.03	$1,550.48	$1,672.86	$1,786.38	$1,549.15

The total cost of claims made by males was also higher than females, comprising 69% of the total cost of claims over the 6 years, and totalling over $966 million NZD, compared to the total cost of $437 million NZD for females. As a result, the cost per claim for males averaged at $1549.15 over the 6-year period; substantially higher than the female cost per claim, which averaged at $887.76 (Table 2).

When we considered specific injury sites, males represented the largest proportion of claims for all sites, except for injuries to the tendons of the finger, thumb and wrist, where females represented 52% of all claims made over the 6-year period (Additional file 1).

### Asian people have had the highest rate of increase in number of claims and Māori maintain the highest cost per claim for tendon/ligament injuries

Over the 6 year study period, people of European ethnicity made up 71% of all claims made, whilst Māori comprised 11.0%, Asian 6%, Pacific peoples 6%, and “Other” ethnicity 6%. This is compared to Aotearoa/New Zealand 2013 Census prioritised ethnicity population figures of 63.7% for European, 15.6% for Māori, 11.7% for Asian, 6.4% for Pacific peoples, 2.5% for “Other” ethnicity (Additional file 1).

This suggests that the total number of claims made by European and “Other” ethnicities were high, relative to their population proportion (over-represented), whilst claims made by Asian and Māori people were low, relative to their population proportion (under-represented). Despite this, the number of claims made by people of Asian ethnicity increased 52% during the 6 year study period (9047 in 2011 to 13,750 in 2016), and 22% by Māori (18,254 in 2011 to 22,187 in 2016). This was a larger rate of increase compared to the 16% elevation in claims overall. In comparison, there was a 13% increase in claims by people of European ethnicity, 12% by Pacific Peoples, and 19% by the “Other” ethnic grouping.

Māori had the highest average cost per claim during the 6 year study period, relative to all other ethnicity groups. In 2016, the average cost per claim for Māori was $1614.05. This was 13% more than the average cost per claim that year, 10% more than the average cost per claim of $1472.11 for people of European ethnicity, and 70% more than the $949.42 for people of Asian ethnicity (Table 3).

**Table 3:** Annual total claim count, total cost and cost per claim for each ethnicity per year within the 6 year study period

End of reporting year	2011	2012	2013	2014	2015	2016	6 Year Total
Total Claim Count							
Asian	9047	9793	10801	11466	12694	13750	67,551
European	123187	128439	130469	130970	137725	139036	789,826
Māori	18254	19422	20195	20344	21636	22187	122,038
Pacific Peoples	10436	11063	11480	11419	11833	11694	67,925
Other	9950	10185	10440	10664	11585	11913	64,737
Total Cost							
Asian	$6,951,815	$8,212,380	$8,514,828	$10,037,482	$12,233,927	$13,054,593	$59,005,025
European	$149,149,851	$151,579,282	$158,871,869	$171,371,660	$193,023,911	$204,676,415	$1,028,672,988
Māori	$25,676,323	$23,906,838	$26,032,065	$28,234,531	$33,027,049	$35,811,028	$172,687,834
Pacific Peoples	$10,133,189	$9,938,416	$10,867,981	$12,171,085	$12,970,137	$14,957,285	$71,038,093
Other	$10,615,297	$10,339,019	$11,430,002	$11,614,411	$13,336,207	$14,835,612	$72,170,548
Average Cost per Claim							
Asian	$768.41	$838.60	$788.34	$875.41	$963.76	$949.42	$863.99
European	$1,210.76	$1,180.17	$1,217.70	$1,308.48	$1,401.52	$1,472.11	$1,298.46
Māori	$1,406.61	$1,230.92	$1,289.04	$1,387.86	$1,526.49	$1,614.05	$1,409.16
Pacific Peoples	$970.98	$898.35	$946.69	$1,065.86	$1,096.10	$1,279.06	$1,042.84
Other	$1,066.86	$1,015.12	$1,094.83	$1,089.12	$1,151.16	$1,245.33	$1,114.83

Tendon injuries in the shoulder were the most commonly claimed injury site for all ethnic groups except Māori, where the knee was the most common claim site (Additional file 1).

**Fig. 2:**
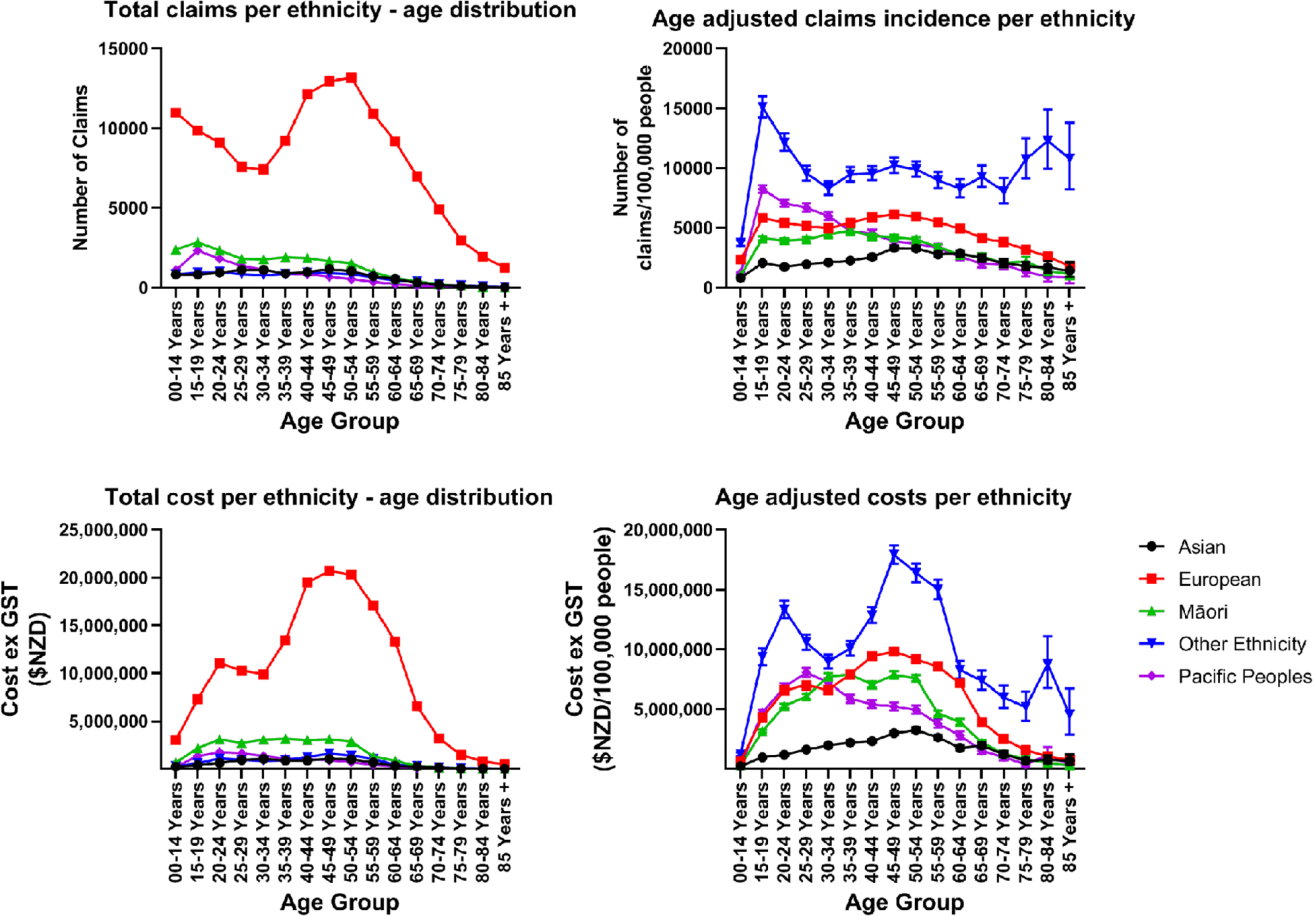
Age distribution of total number of claims and cost of claims made between 2010 and 2016, broken down into each ethnicity, as well as the age-specific incidence for each ethnicity. Error bars represent 95% CI

In an attempt to better understand the ethnic differences within our population, we estimated the age-specific rates for each ethnic group during the census year 2013 using their unique population size as the denominator (Fig. 2).

The “Other” ethnic group had the highest rate and cost of claims of all the ethnic groups. In the younger age groups (< 40 yrs. of age), Pacific Peoples had higher rates of claims than European and Māori, while Asian people had the lowest rate of claims. In the 20–24 age group, for example, the age specific rates per 100,000 people were 12,162 for the “Other” ethnic group, 7062 for Pacific Peoples, 5417 for European people, 3970 for Māori, and 1743 for Asian people. In contrast, European people had higher rates of claims and costs of claims at ages ≥40y, with Māori, Pacific and Asian people having similar rates. In the 50–54 age group, “Other” ethnicities still had the highest rate of claims, at 9906/100,000 people, followed by Europeans with 5947/100,000 people, Māori with 4037/100,000 people, Pacific Peoples 3689/100,000 people, and Asian people 3299/100,000 people.

### Injuries to the shoulder and knee account for the majority of tendon/ligament injuries

Two anatomical sites where injuries occurred more commonly, and had higher costs, were the shoulder and knee. Shoulder injuries accounted for 33% of all claims made over the 6 year period, and 40.6% of all costs, whilst knee injuries accounted for 25% of all claims and 30% of the total cost of all tendon and ligament injuries during this period.

The number of claims at all sites increased substantially over the 6 years, except for tendon and ligament injuries involving the knee. Knee injuries only increased 5% over the 6-year period, which was minor compared to the overall increase of total claims, which was 16.2% during this time period (Table 4).

**Table 4:** Annual total claim count, total cost and cost per claim for each anatomical injury site per year within the 6 year study period

End of reporting year	2011	2012	2013	2014	2015	2016	6 Year Totals
Total Claim Count							
Shoulder	57,586	60,520	60,942	62,727	65,235	64,883	371,893
Elbow	4,577	5,047	6,027	6,071	6,236	5,744	33,702
Knee	45,983	47,971	47,522	44,339	47,313	48,402	281,530
Ankle	16,851	16,978	18,465	19,045	20,179	20,257	111,775
Finger, Thumb and Wrist	31,788	33,333	33,889	35,850	37,904	40,034	212,798
Upper and Lower Arm	1,087	1,168	1,282	1,374	1,615	1,614	8,140
Hip, Upper Leg and Thigh	13,002	13,885	15,258	15,457	16,991	17,646	92,239
Total Cost							
Shoulder	$83,024,989	$84,488,617	$84,022,261	$95,387,639	$108,755,719	$113,599,314	$569,278,539
Elbow	$3,898,729	$3,927,779	$4,649,262	$5,253,332	$6,559,852	$7,127,880	$31,416,834
Knee	$63,619,190	$63,520,525	$68,996,408	$69,660,313	$75,909,954	$81,310,524	$423,016,914
Ankle	$17,253,990	$16,826,440	$19,527,548	$21,029,682	$24,043,240	$25,618,025	$124,298,925
Finger, Thumb and Wrist	$24,778,763	$24,690,955	$26,606,154	$29,293,309	$34,085,529	$38,107,113	$177,561,823
Upper and Lower Arm	$4,249,063	$4,665,164	$4,918,263	$5,175,319	$6,241,071	$7,414,596	$32,663,476
Hip, Upper Leg and Thigh	$5,701,752	$5,856,454	$6,996,852	$7,629,573	$8,995,867	$10,157,480	$45,337,978
Average Cost per Claim							
Shoulder	$1,442.00	$1,396.00	$1,379.00	$1,521.00	$1,667.00	$1,751.00	$1,526.00
Elbow	$851.81	$778.24	$771.41	$865.32	$1,051.93	$1,240.93	$926.61
Knee	$1,383.54	$1,324.14	$1,451.88	$1,571.08	$1,604.42	$1,679.90	$1,502.49
Ankle	$1,023.91	$991.07	$1,057.54	$1,104.21	$1,191.50	$1,264.65	$1,105.48
Finger, Thumb and Wrist	$779.50	$740.74	$785.10	$817.11	$899.26	$951.87	$828.93
Upper and Lower Arm	$3,908.98	$3,994.15	$3,836.40	$3,766.61	$3,864.44	$4,593.93	$3,994.09
Hip, Upper Leg and Thigh	$438.53	$421.78	$458.57	$493.60	$529.45	$575.63	$486.26

### Cost of claims for injuries to all anatomical sites are higher for claimants in their middle years

**Fig. 3:**
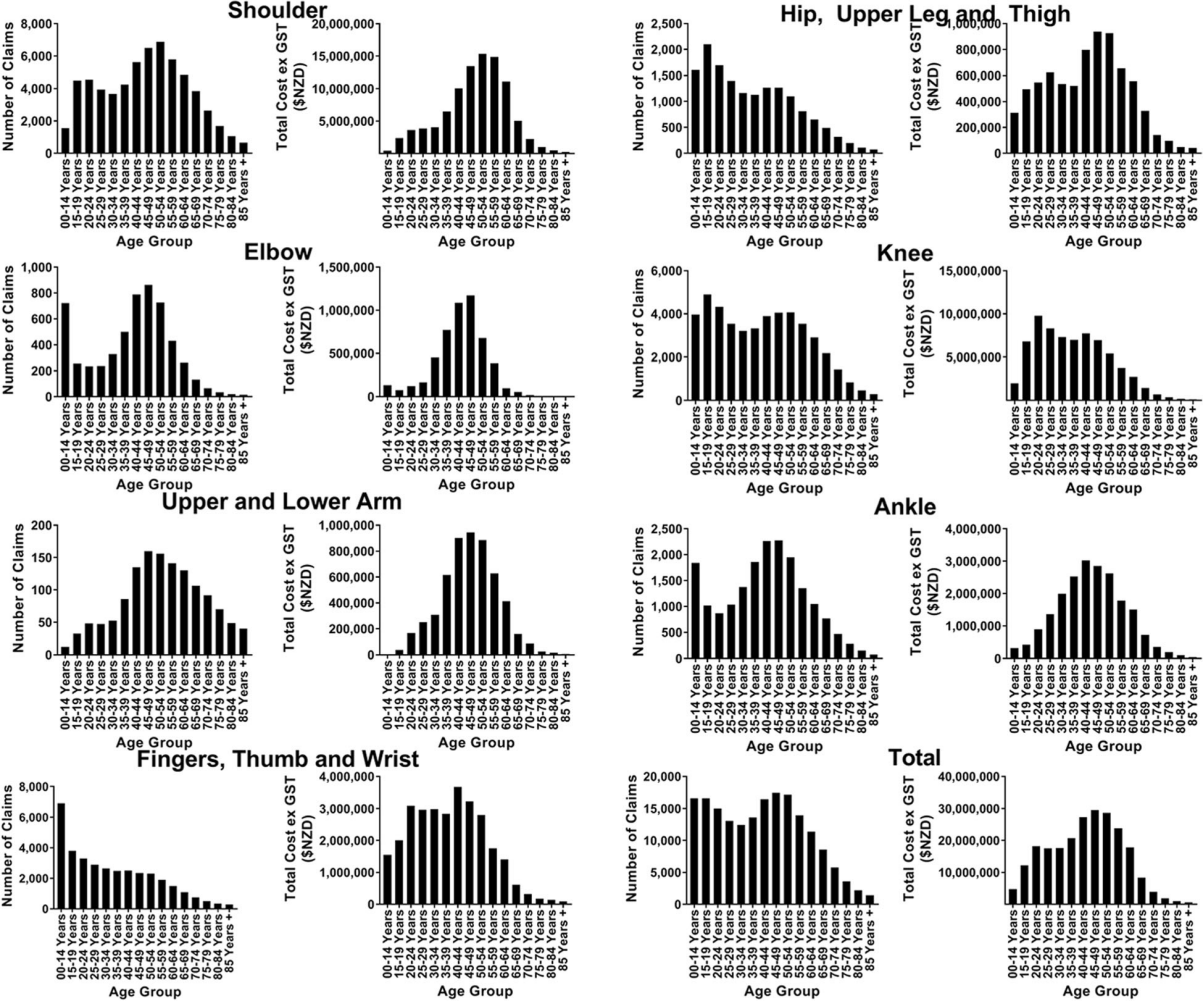
Age distribution of the total number of claims and total cost of claims made between 2010 and 2016, broken down into each anatomical site

The age distribution of claims varied considerably by anatomical site (Fig. 3). Claims made for injuries to the shoulder and upper & lower arm peaked between the ages of 45 and 54. Injuries at the finger, thumb & wrist, hip, upper leg & thigh, and knee appeared to be more common in younger individuals, peaking between the ages of 0 and 19. In comparison, tendon injuries involving the elbow and the ankle showed a bimodal age distribution. Both anatomical sites peak in claims in early life (0–14 years) and peak again in the middle aged population (40–49 years).

When looking at the cost of claims, nearly all injuries cost more for claimants aged 40–54 years, irrespective of the age distribution of the number of claims made, with shoulder injuries making up over half of the costs for these age groups. The exception were tendon injuries of the knee, which peaked in cost between the ages of 20 and 24 (Fig. 3).

## Discussion

This is the first study to demonstrate a rise in tendon and ligament injuries in Aotearoa/New Zealand, both in number of claims and in financial burden at a national level. Furthermore, it, and has identified key epidemiological trends in injuries across different ethnic groups, anatomical sites and age groups. The staggering 1,112,077 claims, at a cost of over $1.4 billion NZD during this study period, in a country with a population of 4,242,048 (2013 census data), clearly highlights these injuries as a significant burden to the Aotearoa/New Zealand healthcare system. To put this into context, during our study period healthcare spending in Aotearoa/New Zealand increased from $13.13 billion NZD in 2010 to $15.63 billion NZD, an increase of 13%. The costs associated with injuries to tendons and ligaments, however, increased by 40%, suggesting that these injuries are increasing in their burden on the Aotearoa/New Zealand healthcare system.

The increasing numbers and costs of specific tendon injuries have been previously highlighted in a number of studies (Clayton & Court-Brown, 2008; Colvin et al., 2012; Paloneva et al., 2015; Sanders Jr. et al., 2015). In contrast, other studies have suggested decreasing rates, specifically of hand and wrist injuries in one region of the United States (de Jong et al., 2014), as well as decreased tendon and ligament injuries of the foot and ankle presenting to emergency departments in the Netherlands (De Boer et al., 2014). Our study found an overall increase in reported tendon and ligament injuries, and although we do not have data on specific injuries, we have identified that these increases in the number and cost of injuries were present for all anatomical sites, including the hand/wrist and the foot/ankle.

The most commonly injured anatomical sites in our study were the shoulder, knee, and finger/hand/wrist, which comprised 33, 25 and 19% of all claims, respectively. In an epidemiological study of 2794 patients presenting to the Orthopaedic Trauma Unit in the Royal Infirmary of Edinburgh over a 5-yr period, meniscal injury of the knee was the most common form of musculoskeletal injury, followed by injuries to the hand extensor tendons and the acromioclavicular joint^.^ (Clayton & Court-Brown, 2008) These injuries are also likely to be contributing to our claims, as are rotator cuff and ACL tears, with rotator cuff repairs increasing 141% from 1996 to 2006 in the US, and increasing 204% from 1998 to 2011 in Finland (Colvin et al., 2012; Paloneva et al., 2015). A recent study in Australia has observed a 143% increase in ACL reconstructions between 2000 and 2015 (Zbrojkiewicz et al., 2018).

Interestingly, injuries to the shoulder were the most common claim made during our study period for all ethnic groups, other than Māori, for whom knee injuries were more common. Whilst the exact reason for this is unknown, a recent study suggested that Māori were under-represented in the number of rotator cuff repairs performed across Aotearoa/New Zealand between March 2009 and December 2010, and that Māori patients who did undergo rotator cuff repair presented younger, with more pain and poorer function than other ethnicities (Maher et al., 2017). The present analysis provides an important addition to the evidence by demonstrating ethnic disparities in the rate and cost of claims made for tendon and ligament injuries. Using the European group as a reference, Pacific peoples had higher claim rates until age 35y, after which Pacific claim rates were lower than those for the European ethnic group. In contrast, Māori and Asian people showed lower claim rates compared with Europeans at every age-group. The age-specific costs of claims for each ethnic group also highlight some interesting patterns, with the cost of claims being similar for Māori, European and Pacific people all showing similar costs up until age 35y, at which point the costs appear to diverge, with European injury claims being the highest and Pacific injury claims having the lowest costs. Meanwhile, the “Other” ethnic grouping had the highest rate of claims and costs at every age-group in this study. It is possible that the estimates for this ethnic grouping are influenced by the low number of claims for this ethnic grouping, and by the differences in the definition used to create the “Other” ethnic grouping between ACC and Statistics NZ. Notwithstanding these potential limitations, this study and others suggests that ethnic inequities in tendon and ligament injuries exist in New Zealand. Although the study was not designed to identify the determinants of these inequities, they likely reflect a range of factors, including age-related differences in the type and severity of injuries, as seen in the aforementioned rotator cuff study (Maher et al., 2017), and barriers to accessing primary care referrals to ACC provider (Jansen et al., 2008). As ACC costs also include covering a component of the patient’s salary during recovery, the higher unemployment rates and lower wages for Māori and Pacific people will also influence the costs associated with injuries in this study.

This analysis has shown that the majority of claims occur during the early years of life (0–19), or during the middle years of life (40–54). Most previous studies looking at specific tendon injuries have observed similar age-related trends (De Boer et al., 2014; de Jong et al., 2014; Gianotti et al., 2009; Houshian et al., 1998; Nyyssonen et al., 2008; Sanders Jr. et al., 2015), the hypothesis being that early injuries are more likely due to sporting injuries and active extracurricular activities, whilst later injuries are more likely to involve a degree of tendon degeneration, predisposing them to injury. Importantly, the highest costs were seen for those in the 40–54 age group. This is the first study to show this trend, and suggests that tendon injuries with a degenerative aspect may be a greater burden to healthcare systems.

This study used certain search terms in an attempt to cover all ACC claims involving tendon and ligament injuries. However, an ACC claim is conditional on the practitioner determining the injury as being caused by an accident, and correctly categorising the injury. Therefore, a limitation of this study is that data encompassing purely degenerative cases of tendon and ligament damage would not be reported, which would result in an underestimation of the true burden. Similarly, if injuries are not be catagorised correctly, this would also underestimate the overall burden. Further problems with this system of reporting and claims come from information bias when a patient self-reports their injury, as issues surrounding recall bias, and accuracy of self-reported information arise.

ACC allow for multiple claims to be made by an individual for one injury, and includes claims made by overseas visitors to Aotearoa/New Zealand. Therefore, injury rates based on ACC claims could overestimate the incidence of injuries during this time-period. To circumvent this, we have calculated direct age-adjusted rates for the year 2013 only, which was when the last census was performed. Census data include all persons in Aotearoa/New Zealand on that day, and thus includes overseas visitors, which gives us the most accurate estimation available.

Using these data to calculate age-adjusted rates for each ethnicity also allows for a more accurate comparison between populations. The New Zealand healthcare system reports prioritized ethnicity, where despite each person being able to identify with multiple ethnicities, the responses are categorized for output in prioritized order (MinistryofHealth, 2017), and this is how ACC report their data also. New Zealand prioritized ethnicity population data, however, is only available during census years and is provided by Stats NZ. Ethnicity may be entered and recorded differently in these databases, and therefore numerator/denominator bias may have been introduced into our estimates. As such, future studies investigating the collection and reporting quality of ethnicity data would be of benefit.

In order to explore our results further, and truly understand the health and socio-economic burden of tendon and ligament injuries, future studies should focus on the prevalence and cost of specific tendon and ligament injury diagnoses, by age group, ethnicity and socioeconomic deprivation, and to calculate rates using specific population denominators similar to the age-adjusted rates reported in Fig. 2. This will help fully characterize the burden of these injuries, identify at risk populations, and tailor intervention and treatments accordingly. Unfortunately, that was not possible within this study due to the anonymized nature of the data we received from ACC and the methods by which they record ethnicities.

A major strength of this study is that it is the first of its kind to demonstrate the burden of all tendon injuries at a national level. Furthermore, it provides a clear insight into how these debilitating injuries are increasing in number and cost, and which injuries and population groups are more likely to be affected. Most published studies on tendon injuries are limited to a smaller sample size and/or a shorter study duration (Albers et al., 2016; Clayton & Court-Brown, 2008; de Jong et al., 2014; Gianotti et al., 2009; Houshian et al., 1998; Nyyssonen et al., 2008; Paloneva et al., 2015; Raikin et al., 2013). However, this study includes data from over 1.1 million claims over a 6 year period, and ACC’s nationwide data collection means we can accumulate comprehensive information regarding the burden of tendon and ligament injuries at a nationwide level. Current reports also suggest that two thirds of tendon and ligament injury cases are not reported (Hopkins et al., 2016), and it is true that this study will also be missing some data due to lack of reporting. However, as ACC offers a “no fault” compensation scheme, reporting of injuries is likely to be more comprehensive than in most other countries.

## Conclusion

Overall, this study has highlighted the increasing number and cost of tendon injuries across Aotearoa/New Zealand; a trend that is likely similar globally. Whilst the data reported here is broad in it’s inclusion, it is hopeful that this can be used as a starting point for framing future studies which more specifically address the growing burden of tendon and ligament injuries. It is difficult to draw many conclusions regarding the reason that these injuries are increasing in number and cost. To our knowledge there have been no changes in how these injuries are diagnosed or treated during this period, and therefore any increases are likely due to behavioural or lifestyle changes in the population, but further studies are needed to address this issue. However, from this data, it is clear that the magnitude of these injuries signifies an urgent need for increased clinical recognition, alongside an improvement in our understanding, treatment and prevention of tendon and ligament injuries.

## Supplementary information

**Additional file 1. **Copy of data received from ACC which was used for this study. Details of claim count per year, and cost of claims per year, broken down into anatomical site of injury, gender of claimant, ethnicity of claimant and age of claimant.

**Additional file 2.** Copy of Aotearoa/New Zealand population by prioritised ethnicity and age groups as of the 2013 census, received from Statistics NZ

**Additional file 3.** List of all tendon/ligament injury diagnoses included in the study

## Data Availability

All data generated or analysed during this study are included in this published article [and its supplementary information files].
