# Supplementary material for: Epidemiology of tendon and ligament injuries in Aotearoa/New Zealand between 2010 and 2016
Source: Inj Epidemiol. 2020 Feb 10;7:5. doi: 10.1186/s40621-020-0231-x (PMC7008565; doi:10.1186/s40621-020-0231-x)
Supplement: Supplementary file 1 — Additional file 1. Copy of data received from ACC which was used for this study. Details of claim count per year, and cost of claims per year, broken down into anatomical site of injury, gender of claimant, ethnicity of claimant and age of claimant. [file 40621_2020_231_MOESM1_ESM.pdf]

Claim count and total costs for claims that received a payment in the 2010/11 financial year, with tendon and ligament injuries, broken down by client gender and the injury site

	Ankle		Elbow		Finger/thumb		Hand/wrist		Hip, Upper Leg, Thigh		Knee		Shoulder (incl Clavicle/blade)		Upper And Lower Arm	
Gender	Claim Count	Cost Ex GST	Claim Count	Cost Ex GST	Claim Count	Cost Ex GST	Claim Count	Cost Ex GST	Claim Count	Cost Ex GST	Claim Count	Cost Ex GST	Claim Count	Cost Ex GST	Claim Count	Cost Ex GST
Female	7780.00	6477057.00	2024.00	1507145.00	711.00	868692.00	15429.00	8706795.00	4655.00	1856830.00	20151.00	21450838.00	22976.00	20014615.00	198.00	460057.00
Male	9071.00	10776933.00	2553.00	2391584.00	1115.00	2316455.00	14533.00	12886821.00	8347.00	3844922.00	25832.00	42168352.00	34610.00	63010374.00	889.00	3789006.00

Claim count and total costs for claims that received a payment in the 2010/11 financial year, with tendon and ligament injuries, broken down by client ethnicity and the injury site

	Ankle		Elbow		Finger/thumb		Hand/wrist		Hip, Upper Leg, Thigh		Knee		Shoulder (incl Clavicle/blade)		Upper And Lower Arm	
Ethnicity Prioritised	Claim Count	Cost Ex GST	Claim Count	Cost Ex GST	Claim Count	Cost Ex GST	Claim Count	Cost Ex GST	Claim Count	Cost Ex GST	Claim Count	Cost Ex GST	Claim Count	Cost Ex GST	Claim Count	Cost Ex GST
Asian	450	$320,462	425	$180,748	99	$168,406	2,310	$1,101,446	496	$177,435	2,369	$2,531,738	2,877	$2,381,662	21	$89,918
European	12,283	$11,695,928	3,139	$2,804,477	1,321	$2,131,730	21,185	$16,014,603	9,489	$4,441,490	31,546	$42,711,362	43,356	$65,778,911	868	$3,571,350
Maori	2,418	$3,388,678	505	$530,757	210	$497,663	3,145	$2,195,494	1,302	$496,942	5,571	$10,000,379	4,989	$8,243,184	114	$323,226
Other Ethnicity	878	$880,756	238	$299,025	101	$188,542	1,734	$1,260,126	836	$307,903	2,763	$3,396,500	3,355	$4,155,217	45	$127,228
Pacific Peoples	822	$968,166	270	$83,722	95	$198,808	1,588	$1,021,947	879	$277,981	3,734	$4,979,211	3,009	$2,466,013	39	$137,341

Claim count and total costs for claims that received a payment in the 2010/11 financial year, with tendon and ligament injuries, broken down by client's age at lodgement (in year bands) and the injury site

	Ankle		Elbow		Finger/thumb		Hand/wrist		Hip, Upper Leg, Thigh		Knee		Shoulder (incl Clavicle/blade)		Upper And Lower Arm	
Age at Lodgement	Claim Count	Cost Ex GST	Claim Count	Cost Ex GST	Claim Count	Cost Ex GST	Claim Count	Cost Ex GST	Claim Count	Cost Ex GST	Claim Count	Cost Ex GST	Claim Count	Cost Ex GST	Claim Count	Cost Ex GST
00-14 Years	1,468	$250,410	889	$161,200	188	$60,970	6,315	$1,279,037	1,336	$271,476	3,613	$1,740,466	1,413	$311,043	1	$45
15-19 Years	901	$300,815	277	$78,641	202	$150,400	3,431	$1,677,321	1,838	$415,782	5,027	$6,579,152	4,262	$2,164,849	20	$17,523
20-24 Years	759	$552,288	196	$154,601	228	$506,175	2,614	$2,160,958	1,467	$395,778	4,477	$8,268,126	4,222	$3,086,719	41	$151,600
25-29 Years	939	$1,116,465	191	$108,197	152	$302,776	2,357	$1,939,775	1,260	$448,626	3,571	$7,364,502	3,579	$3,265,058	40	$217,498
30-34 Years	1,295	$1,630,885	228	$294,977	122	$274,838	2,153	$1,827,895	1,072	$369,632	3,431	$6,946,198	3,379	$3,826,758	49	$225,756
35-39 Years	1,912	$2,571,802	415	$787,391	168	$272,600	2,199	$2,325,698	1,119	$427,519	3,516	$7,108,487	4,153	$6,020,474	81	$448,642
40-44 Years	2,184	$2,442,219	531	$831,447	151	$389,638	2,075	$2,795,301	1,069	$677,252	4,043	$6,810,242	5,378	$9,635,132	94	$581,680
45-49 Years	2,184	$2,588,515	658	$759,894	145	$461,676	2,008	$2,405,081	1,064	$641,464	3,999	$6,244,432	6,276	$11,486,411	141	$824,231
50-54 Years	1,758	$2,018,119	519	$306,892	135	$341,819	1,887	$1,999,148	871	$647,493	3,896	$4,794,381	6,216	$13,074,693	139	$627,212
55-59 Years	1,198	$1,758,633	280	$262,719	99	$152,693	1,452	$1,213,478	628	$482,753	3,270	$3,181,063	5,388	$13,711,325	128	$604,318
60-64 Years	851	$861,794	185	$77,460	90	$163,429	1,207	$1,047,531	493	$401,081	2,634	$2,325,028	4,538	$9,839,198	106	$384,128
65-69 Years	590	$568,712	88	$36,600	58	$30,461	767	$448,886	310	$221,694	1,796	$1,088,961	3,333	$3,427,252	74	$59,761
70-74 Years	393	$286,994	59	$25,386	37	$34,268	567	$203,860	190	$118,458	1,265	$580,500	2,239	$1,636,901	59	$62,327
75-79 Years	214	$137,275	24	$5,518	25	$7,836	369	$107,759	135	$99,496	767	$314,131	1,557	$908,707	49	$24,267
80-84 Years	141	$115,358	21	$4,622	16	$26,215	315	$86,886	97	$37,646	386	$164,454	1,032	$405,225	41	$16,162
85 Years and over	64	$53,707	16	$3,184	10	$9,352	246	$75,000	53	$45,600	292	$109,066	621	$225,244	24	$3,914

Claim count and total costs for claims that received a payment in the 2010/11 financial year, with tendon and ligament injuries, broken down by client gender and ethnicity

	Asian		European		Maori		Other Ethnicity		Pacific Peoples	
Gender	Claim Count	Cost Ex GST	Claim Count	Cost Ex GST	Claim Count	Cost Ex GST	Claim Count	Cost Ex GST	Claim Count	Cost Ex GST
Female	4,217	$2,648,560	55,609	$45,811,127	7,332	$7,754,072	3,900	$2,902,009	2,867	$2,293,332
Male	4,884	$4,385,107	67,696	$103,507,384	11,234	$18,190,816	5,516	$6,990,163	7,619	$8,006,768

Claim count and total costs for claims that received a payment in the 2010/11 financial year, with tendon and ligament injuries, broken down by injury site and ethnicity

	Asian		European		Maori		Other Ethnicity		Pacific Peoples	
Injury Site	Claim Count	Cost Ex GST	Claim Count	Cost Ex GST	Claim Count	Cost Ex GST	Claim Count	Cost Ex GST	Claim Count	Cost Ex GST
Ankle	458	$332,064	12,308	$11,704,849	2,448	$3,413,105	813	$835,464	824	$968,508
Elbow	430	$188,942	3,132	$2,749,494	512	$528,757	225	$308,133	278	$84,900
Finger/thumb	102	$176,832	1,311	$2,133,083	212	$497,685	105	$186,887	97	$198,694
Hand/wrist	2,318	$1,100,258	21,187	$16,074,125	3,203	$2,321,641	1,662	$1,108,266	1,590	$1,016,738
Hip, Upper Leg, Thigh	500	$180,559	9,475	$4,395,336	1,330	$510,107	814	$329,952	884	$287,531
Knee	2,377	$2,544,969	31,594	$42,722,521	5,655	$10,116,953	2,607	$3,196,691	3,742	$4,998,794
Shoulder (incl Clavicle/blade)	2,896	$2,420,156	43,425	$65,976,134	5,092	$8,252,442	3,153	$3,800,832	3,028	$2,578,871
Upper And Lower Arm	20	$89,888	873	$3,562,969	114	$304,195	37	$125,946	43	$166,064

Claim count and total costs for claims that received a payment in the 2010/11 financial year, with tendon and ligament injuries, broken down by client's age at lodgement (in year bands

	Asian		European		Maori		Other Ethnicity		Pacific Peoples	
Age at Lodgement	Claim Count	Cost Ex GST	Claim Count	Cost Ex GST	Claim Count	Cost Ex GST	Claim Count	Cost Ex GST	Claim Count	Cost Ex GST
00-14 Years	705	$195,188	10,522	$2,802,644	2,148	$599,085	846	$203,751	1,004	$275,392
15-19 Years	661	$314,382	9,797	$7,299,927	2,516	$2,001,171	938	$589,979	2,043	$1,148,445
20-24 Years	786	$626,465	8,546	$9,611,286	2,143	$2,700,070	895	$899,666	1,634	$1,466,764
25-29 Years	985	$879,117	7,214	$8,977,877	1,744	$2,653,788	814	$640,570	1,332	$1,604,435
30-34 Years	783	$716,443	7,313	$9,825,308	1,747	$2,764,010	749	$685,794	1,138	$1,367,225
35-39 Years	783	$673,621	9,464	$14,138,394	1,724	$3,018,677	784	$1,068,876	803	$1,059,428
40-44 Years	882	$755,673	11,364	$17,508,371	1,710	$3,641,833	857	$1,278,243	714	$981,414
45-49 Years	1,023	$944,895	12,349	$18,813,815	1,559	$3,304,363	949	$1,450,372	596	$920,305
50-54 Years	867	$656,072	12,091	$19,137,004	1,246	$2,301,043	771	$1,182,948	445	$532,674
55-59 Years	654	$663,401	10,060	$17,826,303	844	$1,506,524	561	$774,010	327	$603,162
60-64 Years	411	$316,200	8,531	$12,879,912	542	$1,020,732	429	$680,351	190	$183,256
65-69 Years	257	$147,262	6,034	$5,132,095	306	$289,630	305	$191,638	115	$122,210
70-74 Years	181	$82,737	4,175	$2,644,963	187	$92,626	195	$111,561	71	$17,338
75-79 Years	82	$45,298	2,785	$1,451,030	87	$37,900	138	$59,116	48	$11,644
80-84 Years	32	$15,130	1,846	$779,431	52	$10,973	102	$45,782	17	$5,252
85 Years and over	9	$1,784	1,214	$490,152	11	$2,463	83	$29,513	9	$1,156

Claim count and total costs for claims that received a payment in the 2011/12 financial year, with tendon and ligament injuries, broken down by client gender and the injury site

	Ankle		Elbow		Finger/thumb		Hand/wrist		Hip, Upper Leg, Thigh		Knee		Shoulder (incl Clavicle/blade)		Upper And Lower Arm	
Gender	Claim Count	Cost Ex GST	Claim Count	Cost Ex GST	Claim Count	Cost Ex GST	Claim Count	Cost Ex GST	Claim Count	Cost Ex GST	Claim Count	Cost Ex GST	Claim Count	Cost Ex GST	Claim Count	Cost Ex GST
Female	7892.00	5632890.00	2142.00	1335484.00	796.00	1037780.00	16133.00	9004547.00	4931.00	1852595.00	21116.00	21621419.00	24373.00	21533947.00	235.00	477587.00
Male	9086.00	11193550.00	2905.00	2592295.00	1156.00	2061827.00	15248.00	12586801.00	8954.00	4003859.00	26855.00	41899106.00	36147.00	62954670.00	933.00	4187577.00

Claim count and total costs for claims that received a payment in the 2011/12 financial year, with tendon and ligament injuries, broken down by client ethnicity and the injury site

	Ankle		Elbow		Finger/thumb		Hand/wrist		Hip, Upper Leg, Thigh		Knee		Shoulder (incl Clavicle/blade)		Upper And Lower Arm	
Ethnicity Prioritised	Claim Count	Cost Ex GST	Claim Count	Cost Ex GST	Claim Count	Cost Ex GST	Claim Count	Cost Ex GST	Claim Count	Cost Ex GST	Claim Count	Cost Ex GST	Claim Count	Cost Ex GST	Claim Count	Cost Ex GST
Asian	478	$448,338	416	$169,654	96	$162,261	2,524	$1,338,359	577	$176,038	2,523	$2,979,133	3,155	$2,872,426	24	$66,171
European	12,248	$11,603,473	3,490	$2,795,008	1,431	$2,233,225	21,965	$15,957,714	10,120	$4,747,189	32,858	$43,026,950	45,406	$67,512,021	921	$3,703,702
Maori	2,500	$3,186,760	531	$505,154	219	$309,488	3,402	$2,292,738	1,338	$442,055	5,851	$9,036,204	5,450	$7,523,574	131	$610,865
Other Ethnicity	885	$745,731	302	$355,854	122	$190,213	1,771	$1,119,037	888	$261,969	2,756	$3,778,131	3,411	$3,761,676	50	$126,408
Pacific Peoples	867	$842,139	308	$102,109	84	$204,421	1,719	$883,500	962	$229,203	3,983	$4,700,107	3,098	$2,818,919	42	$158,018

Claim count and total costs for claims that received a payment in the 2011/12 financial year, with tendon and ligament injuries, broken down by client's age at lodgement (in year bands) and the injury site

	Ankle		Elbow		Finger/thumb		Hand/wrist		Hip, Upper Leg, Thigh		Knee		Shoulder (incl Clavicle/blade)		Upper And Lower Arm	
Age at Lodgement	Claim Count	Cost Ex GST	Claim Count	Cost Ex GST	Claim Count	Cost Ex GST	Claim Count	Cost Ex GST	Claim Count	Cost Ex GST	Claim Count	Cost Ex GST	Claim Count	Cost Ex GST	Claim Count	Cost Ex GST
00-14 Years	1,675	$271,115	883	$170,222	228	$77,097	6,525	$1,395,284	1,550	$285,052	4,109	$1,949,372	1,461	$405,519	6	$2,186
15-19 Years	873	$385,790	252	$61,983	194	$192,627	3,524	$1,462,777	1,969	$442,632	5,313	$6,317,317	4,521	$2,232,403	24	$60,989
20-24 Years	771	$566,358	239	$104,204	235	$292,872	2,987	$1,937,998	1,590	$497,541	4,672	$8,507,417	4,461	$3,045,524	39	$93,862
25-29 Years	919	$911,699	213	$133,489	160	$263,623	2,382	$2,180,392	1,293	$516,172	3,682	$7,393,283	3,630	$3,447,392	42	$284,383
30-34 Years	1,359	$1,864,158	288	$279,975	142	$292,331	2,213	$2,191,829	1,046	$430,636	3,336	$6,049,621	3,507	$3,588,559	40	$207,803
35-39 Years	1,854	$2,204,233	462	$740,406	175	$272,115	2,237	$2,015,097	1,095	$416,748	3,561	$6,657,990	4,271	$5,967,960	85	$536,215
40-44 Years	2,079	$2,163,619	635	$781,943	167	$486,592	2,185	$2,629,365	1,187	$587,968	4,035	$7,322,914	5,572	$9,900,481	116	$764,284
45-49 Years	2,195	$2,536,896	713	$962,476	150	$463,656	1,997	$2,383,664	1,106	$772,577	4,139	$6,472,372	6,395	$12,130,351	147	$882,176
50-54 Years	1,699	$2,108,764	592	$386,988	152	$383,986	1,947	$2,163,629	962	$636,356	4,118	$4,852,116	6,812	$14,239,131	132	$785,478
55-59 Years	1,230	$1,586,502	337	$161,674	111	$152,950	1,566	$1,363,457	640	$504,167	3,349	$3,175,805	5,694	$12,541,153	124	$655,675
60-64 Years	930	$1,082,626	225	$70,192	88	$132,966	1,326	$955,634	540	$374,247	2,863	$2,550,125	4,802	$9,329,961	114	$266,949
65-69 Years	598	$572,973	107	$40,547	56	$35,896	883	$386,186	374	$176,664	1,983	$1,084,736	3,420	$4,140,102	86	$49,030
70-74 Years	370	$241,490	46	$18,455	42	$26,326	654	$222,526	239	$69,400	1,281	$543,375	2,619	$1,955,803	74	$43,920
75-79 Years	240	$215,171	22	$2,735	21	$8,093	427	$156,242	136	$70,836	784	$354,731	1,653	$858,974	60	$15,834
80-84 Years	120	$65,738	20	$5,051	20	$16,641	285	$87,356	90	$30,571	465	$169,510	1,081	$441,577	43	$9,864
85 Years and over	66	$49,308	13	$7,439	11	$1,838	243	$59,910	68	$44,887	281	$119,842	621	$263,726	36	$6,518

Claim count and total costs for claims that received a payment in the 2011/12 financial year, with tendon and ligament injuries, broken down by client gender and ethnicity

	Asian		European		Maori		Other Ethnicity		Pacific Peoples	
Gender	Claim Count	Cost Ex GST	Claim Count	Cost Ex GST	Claim Count	Cost Ex GST	Claim Count	Cost Ex GST	Claim Count	Cost Ex GST
Female	4,479	$3,016,056	57,953	$47,103,181	7,973	$7,072,446	4,023	$2,972,166	3,194	$2,374,914
Male	5,366	$5,269,013	70,528	$104,113,314	11,776	$17,346,111	5,683	$6,917,787	7,931	$7,764,334

Claim count and total costs for claims that received a payment in the 2011/12 financial year, with tendon and ligament injuries, broken down by injury site and ethnicity

	Asian		European		Maori		Other Ethnicity		Pacific Peoples	
Injury Site	Claim Count	Cost Ex GST	Claim Count	Cost Ex GST	Claim Count	Cost Ex GST	Claim Count	Cost Ex GST	Claim Count	Cost Ex GST
Ankle	482	$459,547	12,241	$11,548,320	2,543	$3,235,111	848	$745,078	866	$839,215
Elbow	423	$170,789	3,488	$2,718,392	544	$505,441	282	$367,344	309	$102,359
Finger/thumb	96	$162,385	1,431	$2,220,082	224	$340,337	116	$172,838	85	$203,965
Hand/wrist	2,533	$1,338,524	21,933	$15,888,136	3,463	$2,435,785	1,724	$1,041,324	1,732	$926,601
Hip, Upper Leg, Thigh	576	$174,891	10,122	$4,667,567	1,370	$445,330	846	$340,766	973	$231,305
Knee	2,538	$2,994,348	32,874	$42,786,159	5,928	$9,267,639	2,626	$3,650,220	4,000	$4,770,128
Shoulder (incl Clavicle/blade)	3,175	$2,918,724	45,466	$67,691,547	5,548	$7,585,591	3,219	$3,457,115	3,114	$2,881,256
Upper And Lower Arm	22	$65,862	926	$3,696,292	129	$603,324	45	$115,268	46	$184,419

Claim count and total costs for claims that received a payment in the 2011/12 financial year, with tendon and ligament injuries, broken down by client's age at lodgement (in year bands

	Asian		European		Maori		Other Ethnicity		Pacific Peoples	
Age at Lodgement	Claim Count	Cost Ex GST	Claim Count	Cost Ex GST	Claim Count	Cost Ex GST	Claim Count	Cost Ex GST	Claim Count	Cost Ex GST
00-14 Years	798	$233,818	11,284	$3,120,994	2,348	$642,799	851	$241,966	1,157	$317,127
15-19 Years	723	$394,852	10,032	$7,171,635	2,741	$1,813,282	974	$534,689	2,197	$1,225,222
20-24 Years	866	$673,082	9,105	$9,363,608	2,218	$2,473,542	1,013	$1,090,447	1,797	$1,474,064
25-29 Years	1,039	$1,010,835	7,369	$9,569,960	1,697	$2,171,542	861	$937,438	1,357	$1,438,957
30-34 Years	890	$886,988	7,302	$9,327,641	1,828	$2,669,727	784	$710,032	1,128	$1,217,090
35-39 Years	867	$819,586	9,477	$13,074,875	1,788	$2,856,978	796	$909,238	811	$1,156,239
40-44 Years	983	$846,303	11,591	$18,486,305	1,809	$3,216,020	889	$1,245,519	706	$840,856
45-49 Years	1,002	$1,022,267	12,635	$20,113,942	1,632	$3,296,592	922	$1,295,376	650	$913,781
50-54 Years	934	$952,289	12,790	$20,451,259	1,443	$2,561,792	764	$969,365	482	$617,118
55-59 Years	709	$725,379	10,471	$16,670,796	912	$1,569,427	613	$692,915	346	$509,158
60-64 Years	447	$376,442	9,211	$12,587,810	585	$758,338	425	$783,823	220	$259,136
65-69 Years	276	$206,789	6,410	$5,695,640	351	$232,545	333	$235,775	138	$115,601
70-74 Years	178	$74,183	4,673	$2,788,852	216	$99,200	190	$120,253	67	$37,860
75-79 Years	87	$44,033	2,971	$1,526,243	117	$40,586	132	$51,479	35	$10,249
80-84 Years	37	$15,564	1,924	$766,626	45	$12,006	91	$26,072	27	$6,039
85 Years and over	9	$2,662	1,236	$500,309	19	$4,180	68	$45,565	7	$752

Claim count and total costs for claims that received a payment in the 2012/13 financial year, with tendon and ligament injuries, broken down by client gender and the injury site

	Ankle		Elbow		Finger/thumb		Hand/wrist		Hip, Upper Leg, Thigh		Knee		Shoulder (incl Clavicle/blade)		Upper And Lower Arm	
Gender	Claim Count	Cost Ex GST	Claim Count	Cost Ex GST	Claim Count	Cost Ex GST	Claim Count	Cost Ex GST	Claim Count	Cost Ex GST	Claim Count	Cost Ex GST	Claim Count	Cost Ex GST	Claim Count	Cost Ex GST
Female	8599.00	6796381.00	2569.00	1425523.00	914.00	1099940.00	16542.00	10091477.00	5524.00	2390860.00	21114.00	23676175.00	24699.00	21823928.00	253.00	668947.00
Male	9866.00	12731167.00	3458.00	3223739.00	1242.00	2254926.00	15191.00	13159811.00	9734.00	4605992.00	26408.00	45320233.00	36243.00	62198333.00	1029.00	4249316.00

Claim count and total costs for claims that received a payment in the 2012/13 financial year, with tendon and ligament injuries, broken down by client ethnicity and the injury site

	Ankle		Elbow		Finger/thumb		Hand/wrist		Hip, Upper Leg, Thigh		Knee		Shoulder (incl Clavicle/blade)		Upper And Lower Arm	
Ethnicity Prioritised	Claim Count	Cost Ex GST	Claim Count	Cost Ex GST	Claim Count	Cost Ex GST	Claim Count	Cost Ex GST	Claim Count	Cost Ex GST	Claim Count	Cost Ex GST	Claim Count	Cost Ex GST	Claim Count	Cost Ex GST
Asian	501	$583,170	535	$237,738	128	$106,650	2,783	$1,367,036	739	$252,227	2,680	$3,120,823	3,412	$2,763,493	23	$83,691
European	13,310	$13,558,933	4,215	$3,487,838	1,522	$2,316,342	22,115	$17,201,550	10,868	$5,428,859	32,218	$46,051,926	45,223	$66,983,122	998	$3,843,299
Maori	2,718	$3,523,243	589	$445,672	264	$442,379	3,418	$2,351,516	1,589	$612,456	6,014	$10,495,750	5,451	$7,574,534	152	$586,515
Other Ethnicity	1,017	$862,929	356	$411,873	131	$312,344	1,719	$1,403,041	978	$389,424	2,659	$3,925,101	3,519	$3,820,731	61	$304,559
Pacific Peoples	919	$999,273	332	$66,141	111	$177,150	1,698	$928,144	1,084	$313,886	3,951	$5,402,807	3,337	$2,880,381	48	$100,199
Residual Categories	430	$307,975	109	$64,829	37	$62,630	445	$324,899	389	$155,916	808	$1,246,083	1,275	$1,203,383	22	$59,549

Claim count and total costs for claims that received a payment in the 2012/13 financial year, with tendon and ligament injuries, broken down by client's age at lodgement (in year bands) and the injury site

	Ankle		Elbow		Finger/thumb		Hand/wrist		Hip, Upper Leg, Thigh		Knee		Shoulder (incl Clavicle/blade)		Upper And Lower Arm	
Age at Lodgement	Claim Count	Cost Ex GST	Claim Count	Cost Ex GST	Claim Count	Cost Ex GST	Claim Count	Cost Ex GST	Claim Count	Cost Ex GST	Claim Count	Cost Ex GST	Claim Count	Cost Ex GST	Claim Count	Cost Ex GST
00-14 Years	1,752	$275,830	740	$114,311	200	$53,441	6,381	$1,364,497	1,663	$283,510	3,896	$1,945,374	1,507	$395,264	5	$1,914
15-19 Years	987	$394,848	266	$81,996	223	$187,168	3,535	$1,670,554	2,069	$495,929	5,145	$6,761,360	4,546	$2,162,634	39	$69,561
20-24 Years	846	$1,075,389	230	$100,156	217	$348,915	2,931	$2,227,624	1,736	$460,888	4,628	$10,212,196	4,571	$3,039,167	44	$192,231
25-29 Years	940	$1,115,660	234	$105,070	201	$350,282	2,427	$2,108,478	1,357	$566,114	3,644	$8,336,099	3,862	$3,708,877	47	$186,337
30-34 Years	1,417	$1,936,491	325	$356,302	187	$320,807	2,335	$2,262,053	1,199	$546,304	3,144	$7,141,112	3,578	$3,506,046	38	$242,409
35-39 Years	1,910	$2,148,916	545	$658,803	186	$369,673	2,165	$2,324,935	1,161	$500,815	3,456	$6,901,947	4,210	$6,051,189	84	$534,587
40-44 Years	2,358	$2,945,687	897	$996,722	197	$623,208	2,266	$2,895,078	1,272	$715,496	4,048	$7,551,422	5,607	$8,943,528	139	$1,040,761
45-49 Years	2,283	$2,952,666	969	$1,138,897	182	$404,456	2,084	$2,562,380	1,263	$957,352	4,083	$6,546,227	6,356	$12,072,568	158	$825,774
50-54 Years	1,992	$2,524,560	772	$561,571	170	$294,332	1,999	$2,219,884	1,065	$864,139	4,190	$5,133,865	6,784	$14,001,970	149	$737,079
55-59 Years	1,321	$1,573,534	467	$377,109	124	$135,296	1,704	$1,534,188	742	$621,782	3,564	$3,332,825	5,573	$12,307,443	118	$590,265
60-64 Years	1,015	$1,301,484	318	$88,209	103	$158,603	1,291	$1,026,402	626	$401,907	2,776	$2,473,838	4,743	$9,571,509	126	$190,877
65-69 Years	715	$595,770	126	$35,285	69	$47,734	966	$496,262	479	$307,578	2,047	$1,321,721	3,703	$4,622,567	93	$144,197
70-74 Years	434	$341,732	69	$16,856	48	$23,867	644	$246,235	295	$104,693	1,406	$681,855	2,616	$2,122,054	87	$125,012
75-79 Years	250	$209,074	36	$7,562	23	$9,685	406	$120,367	183	$69,199	778	$314,306	1,643	$887,213	72	$18,854
80-84 Years	170	$91,760	16	$3,969	21	$25,673	326	$85,946	84	$56,193	451	$210,539	1,016	$413,559	51	$11,051
85 Years and over	75	$44,146	17	$6,444	5	$1,726	273	$106,405	64	$44,955	266	$131,719	627	$216,673	32	$7,354

Claim count and total costs for claims that received a payment in the 2012/13 financial year, with tendon and ligament injuries, broken down by client gender and ethnicity

	Asian		European		Maori		Other Ethnicity		Pacific Peoples	
Gender	Claim Count	Cost Ex GST	Claim Count	Cost Ex GST	Claim Count	Cost Ex GST	Claim Count	Cost Ex GST	Claim Count	Cost Ex GST
Female	4,822	$3,257,021	59,436	$50,422,526	8,433	$8,217,274	4,141	$3,450,229	3,385	$2,642,805
Male	5,984	$5,237,744	71,162	$108,176,495	12,103	$18,434,211	5,781	$7,591,824	8,139	$8,261,636

Claim count and total costs for claims that received a payment in the 2012/13 financial year, with tendon and ligament injuries, broken down by injury site and ethnicity

	Asian		European		Maori		Other Ethnicity		Pacific Peoples	
Injury Site	Claim Count	Cost Ex GST	Claim Count	Cost Ex GST	Claim Count	Cost Ex GST	Claim Count	Cost Ex GST	Claim Count	Cost Ex GST
Ankle	502	$582,996	13,311	$13,549,674	2,762	$3,571,756	969	$825,160	923	$998,657
Elbow	538	$239,907	4,227	$3,424,661	598	$452,436	331	$425,128	332	$65,686
Finger/thumb	129	$109,865	1,526	$2,341,041	267	$443,450	121	$282,765	113	$177,745
Hand/wrist	2,786	$1,368,727	22,120	$17,057,079	3,473	$2,557,128	1,654	$1,365,629	1,703	$926,597
Hip, Upper Leg, Thigh	736	$251,278	10,874	$5,475,745	1,629	$620,983	936	$340,537	1,085	$313,074
Knee	2,680	$3,119,911	32,235	$45,782,420	6,078	$10,772,383	2,543	$3,845,753	3,980	$5,435,490
Shoulder (incl Clavicle/blade)	3,413	$2,738,489	45,309	$67,150,318	5,573	$7,622,424	3,311	$3,652,086	3,337	$2,886,527
Upper And Lower Arm	22	$83,592	996	$3,818,082	156	$610,925	57	$304,998	51	$100,666

Claim count and total costs for claims that received a payment in the 2012/13 financial year, with tendon and ligament injuries, broken down by client's age at lodgement (in year bands

	Asian		European		Maori		Other Ethnicity		Pacific Peoples	
Age at Lodgement	Claim Count	Cost Ex GST	Claim Count	Cost Ex GST	Claim Count	Cost Ex GST	Claim Count	Cost Ex GST	Claim Count	Cost Ex GST
00-14 Years	816	$235,683	10,998	$3,023,915	2,407	$673,164	831	$275,288	1,093	$226,451
15-19 Years	814	$386,596	9,862	$7,333,201	2,854	$2,179,886	950	$588,060	2,328	$1,334,859
20-24 Years	941	$644,544	9,104	$11,044,845	2,338	$3,103,775	1001	$1,099,064	1,822	$1,767,736
25-29 Years	1,116	$918,768	7,563	$10,274,944	1,821	$2,714,065	848	$939,177	1,366	$1,649,265
30-34 Years	1,117	$1,046,688	7,422	$9,909,891	1,776	$3,071,724	779	$841,273	1,129	$1,368,271
35-39 Years	890	$864,504	9,201	$13,454,456	1,918	$3,185,376	850	$904,698	857	$1,070,177
40-44 Years	985	$887,956	12,165	$19,531,457	1,853	$3,027,456	940	$1,263,907	843	$1,011,787
45-49 Years	1,168	$1,053,608	12,939	$20,717,540	1,669	$3,115,311	921	1615491	680	$915,796
50-54 Years	1,030	$1,019,441	13,189	$20,334,647	1,524	$2,865,113	847	$1,404,053	532	$714,178
55-59 Years	721	$682,791	10,921	$17,066,163	973	$1,330,185	626	$1,046,920	368	$420,171
60-64 Years	544	$332,084	9,176	$13,333,153	597	$850,928	449	$447,985	233	$252,562
65-69 Years	309	$243,079	6,976	$6,590,640	395	$330,732	388	$308,310	130	$98,354
70-74 Years	178	$105,873	4,918	$3,220,527	210	$131,590	207	$153,847	85	$43,419
75-79 Years	104	$39,884	2,973	$1,466,761	134	$49,794	144	$69,499	36	$10,321
80-84 Years	50	$22,159	1,938	$779,759	47	$17,708	86	$61,286	14	$17,780
85 Years and over	23	$11,107	1,253	$517,125	20	$4,677	55	$23,197	8	$3,314

Claim count and total costs for claims that received a payment in the 2013/14 financial year, with tendon and ligament injuries, broken down by client gender and the injury site

	Ankle		Elbow		Finger/thumb		Hand/wrist		Hip, Upper Leg, Thigh		Knee		Shoulder (incl Clavicle/blade)		Upper And Lower Arm	
Gender	Claim Count	Cost Ex GST	Claim Count	Cost Ex GST	Claim Count	Cost Ex GST	Claim Count	Cost Ex GST	Claim Count	Cost Ex GST	Claim Count	Cost Ex GST	Claim Count	Cost Ex GST	Claim Count	Cost Ex GST
Female	8912.00	7538349.00	2558.00	1698439.00	1122.00	1370821.00	17322.00	10769464.00	5661.00	2686863.00	19952.00	24314635.00	25911.00	24295379.00	274.00	821862.00
Male	10133.00	13491333.00	3513.00	3554893.00	1535.00	2900131.00	15871.00	14252893.00	9796.00	4942710.00	24387.00	45345678.00	36816.00	71092260.00	1100.00	4353457.00

Claim count and total costs for claims that received a payment in the 2013/14 financial year, with tendon and ligament injuries, broken down by client ethnicity and the injury site

	Ankle		Elbow		Finger/thumb		Hand/wrist		Hip, Upper Leg, Thigh		Knee		Shoulder (incl Clavicle/blade)		Upper And Lower Arm	
Ethnicity Prioritised	Claim Count	Cost Ex GST	Claim Count	Cost Ex GST	Claim Count	Cost Ex GST	Claim Count	Cost Ex GST	Claim Count	Cost Ex GST	Claim Count	Cost Ex GST	Claim Count	Cost Ex GST	Claim Count	Cost Ex GST
Asian	584	$717,738	535	$278,992	146	$171,770	3,076	$1,519,369	748	$265,416	2,736	$3,531,608	3,605	$3,350,487	36	$202,102
European	13,728	$14,462,754	4,291	$3,794,759	1,878	$3,012,684	22,918	$18,367,077	10,904	$5,947,812	29,676	$46,017,261	46,513	$75,818,953	1,062	$3,950,360
Maori	2,716	$3,633,869	579	$670,643	334	$587,622	3,640	$2,714,238	1,624	$699,660	5,643	$10,348,555	5,651	$8,879,454	157	$700,490
Other Ethnicity	1,056	$1,053,102	370	$372,971	157	$234,951	1,813	$1,387,196	1,063	$404,437	2,450	$3,784,896	3,686	$4,164,549	69	$212,309
Pacific Peoples	961	$1,162,219	296	$135,967	142	$263,926	1,746	$1,034,476	1,118	$312,248	3,834	$5,977,993	3,272	$3,174,197	50	$110,059

Claim count and total costs for claims that received a payment in the 2013/14 financial year, with tendon and ligament injuries, broken down by client's age at lodgement (in year bands) and the injury site

	Ankle		Elbow		Finger/thumb		Hand/wrist		Hip, Upper Leg, Thigh		Knee		Shoulder (incl Clavicle/blade)		Upper And Lower Arm	
Age at Lodgement	Claim Count	Cost Ex GST	Claim Count	Cost Ex GST	Claim Count	Cost Ex GST	Claim Count	Cost Ex GST	Claim Count	Cost Ex GST	Claim Count	Cost Ex GST	Claim Count	Cost Ex GST	Claim Count	Cost Ex GST
00-14 Years	2,006	$335,367	622	$117,664	258	$72,182	6,616	$1,478,447	1,633	$339,206	3,785	$1,936,623	1,625	$399,252	13	$3,559
15-19 Years	1,063	$378,556	242	$96,775	260	$284,543	3,583	$1,768,874	2,224	$529,288	4,719	$6,797,145	4,548	$2,569,960	29	$17,418
20-24 Years	913	$1,021,199	231	$71,045	275	$550,056	3,056	$2,474,695	1,739	$645,630	4,063	$9,895,422	4,789	$3,557,624	46	$113,046
25-29 Years	1,003	$1,226,017	248	$108,594	227	$407,060	2,611	$2,542,998	1,370	$736,844	3,432	$7,907,957	4,057	$3,922,594	48	$182,307
30-34 Years	1,367	$1,803,126	367	$413,850	207	$353,973	2,510	$2,496,030	1,164	$501,572	3,073	$7,159,552	3,798	$3,943,715	52	$377,500
35-39 Years	1,842	$2,383,519	546	$700,175	211	$399,993	2,289	$2,517,655	1,105	$563,161	3,136	$6,612,366	4,182	$6,275,101	88	$644,757
40-44 Years	2,362	$3,302,736	904	$1,121,122	214	$734,062	2,356	$3,049,852	1,276	$854,909	3,674	$8,213,305	5,690	$10,108,253	148	$941,413
45-49 Years	2,255	$3,102,397	968	$1,222,161	244	$570,317	2,130	$2,604,730	1,284	$803,643	3,781	$6,663,783	6,467	$13,386,007	163	$854,473
50-54 Years	1,988	$2,792,752	870	$860,828	216	$315,172	2,155	$2,411,602	1,047	$957,246	3,767	$5,144,151	6,939	$15,349,931	156	$880,466
55-59 Years	1,379	$1,807,570	491	$364,106	180	$270,874	1,760	$1,345,831	822	$570,085	3,344	$3,832,979	5,832	$15,383,913	150	$471,259
60-64 Years	1,078	$1,510,142	271	$90,032	133	$126,162	1,348	$1,149,382	643	$563,351	2,674	$2,602,543	4,925	$11,448,569	140	$440,411
65-69 Years	787	$732,242	157	$53,484	110	$102,556	1,045	$556,305	472	$275,814	2,093	$1,491,459	3,992	$5,043,667	99	$92,285
70-74 Years	481	$348,610	78	$17,570	63	$45,337	689	$252,247	324	$107,132	1,342	$719,164	2,626	$2,287,638	97	$101,076
75-79 Years	285	$181,496	36	$7,167	28	$22,375	458	$157,560	186	$81,570	740	$345,680	1,599	$1,029,118	66	$19,863
80-84 Years	153	$74,298	27	$6,675	20	$10,323	332	$141,345	104	$39,468	427	$199,460	1,014	$429,358	43	$27,365
85 Years and over	83	$29,656	13	$2,085	11	$5,966	255	$74,805	64	$60,655	289	$138,722	644	$252,941	36	$8,121

Claim count and total costs for claims that received a payment in the 2013/14 financial year, with tendon and ligament injuries, broken down by client gender and ethnicity

	Asian		European		Maori		Other Ethnicity		Pacific Peoples	
Gender	Claim Count	Cost Ex GST	Claim Count	Cost Ex GST	Claim Count	Cost Ex GST	Claim Count	Cost Ex GST	Claim Count	Cost Ex GST
Female	5,113	$3,757,001	60,271	$54,843,101	8,632	$8,782,558	4,180	$3,243,566	3,522	$2,957,033
Male	6,373	$6,301,398	70,814	$116,432,866	12,033	$20,076,876	5,968	$7,897,062	7,968	$9,174,587

Claim count and total costs for claims that received a payment in the 2013/14 financial year, with tendon and ligament injuries, broken down by injury site and ethnicity

	Asian		European		Maori		Other Ethnicity		Pacific Peoples	
Injury Site	Claim Count	Cost Ex GST	Claim Count	Cost Ex GST	Claim Count	Cost Ex GST	Claim Count	Cost Ex GST	Claim Count	Cost Ex GST
Ankle	579	$713,775	13,736	$14,456,591	2,748	$3,654,829	1,017	$1,063,492	969	$1,142,460
Elbow	535	$278,064	4,299	$3,717,699	582	$670,057	354	$412,404	299	$136,223
Finger/thumb	144	$170,045	1,876	$3,023,395	347	$603,148	150	$231,662	142	$243,395
Hand/wrist	3,093	$1,522,296	22,891	$18,422,871	3,719	$2,822,812	1,732	$1,319,817	1,756	$979,053
Hip, Upper Leg, Thigh	753	$273,575	10,919	$5,879,536	1,652	$693,881	1,004	$496,771	1,132	$316,567
Knee	2,738	$3,547,050	29,717	$46,004,816	5,691	$10,577,903	2,337	$3,587,302	3,862	$5,973,422
Shoulder (incl Clavicle/blade)	3,609	$3,351,583	46,584	$75,816,754	5,767	$9,136,013	3,488	$3,821,115	3,279	$3,230,350
Upper And Lower Arm	35	$202,011	1,063	$3,954,304	159	$700,792	66	$208,063	51	$110,149

Claim count and total costs for claims that received a payment in the 2013/14 financial year, with tendon and ligament injuries, broken down by client's age at lodgement (in year bands

	Asian		European		Maori		Other Ethnicity		Pacific Peoples	
Age at Lodgement	Claim Count	Cost Ex GST	Claim Count	Cost Ex GST	Claim Count	Cost Ex GST	Claim Count	Cost Ex GST	Claim Count	Cost Ex GST
00-14 Years	861	$231,682	11,148	$3,128,412	2,538	$756,992	864	$247,365	1,148	$318,816
15-19 Years	797	$502,768	9,708	$7,681,626	2,874	$2,151,512	953	$523,690	2,335	$1,584,710
20-24 Years	977	$799,010	9,118	$11,391,254	2,218	$3,148,661	991	$1,046,939	1,809	$1,926,875
25-29 Years	1,195	$1,108,982	7,696	$10,626,091	1,839	$2,813,708	915	$914,997	1,350	$1,594,488
30-34 Years	1,260	$1,130,661	7,451	$10,274,958	1,902	$3,120,806	833	$801,370	1,093	$1,616,528
35-39 Years	957	$896,327	8,928	$13,598,545	1,798	$3,363,912	816	$1,254,316	900	$1,005,457
40-44 Years	1,020	$1,234,070	12,018	$21,275,585	1,852	$3,163,514	951	$1,495,558	786	$1,176,062
45-49 Years	1,111	$1,283,327	12,871	$22,056,276	1,736	$3,520,677	915	$1,456,868	659	$883,053
50-54 Years	1,133	$1,080,414	13,192	$22,365,865	1,451	$3,256,233	873	$1,239,975	493	$794,823
55-59 Years	799	$776,417	11,096	$19,846,547	1,004	$1,965,877	677	$847,080	384	$678,441
60-64 Years	595	$562,636	9,197	$15,187,353	685	$1,136,363	489	$657,403	247	$391,511
65-69 Years	346	$214,846	7,553	$7,402,860	352	$244,906	369	$396,628	135	$88,573
70-74 Years	215	$132,077	4,959	$3,458,451	229	$139,298	216	$125,939	81	$23,009
75-79 Years	140	$68,489	2,962	$1,634,535	127	$63,920	133	$67,176	36	$10,709
80-84 Years	55	$23,908	1,909	$822,772	37	$9,817	91	$35,033	28	$36,764
85 Years and over	25	$12,782	1,279	$524,837	23	$3,238	62	$30,291	6	$1,801

Claim count and total costs for claims that received a payment in the 2014/15 financial year, with tendon and ligament injuries, broken down by client gender and the injury site

	Ankle		Elbow		Finger/thumb		Hand/wrist		Hip, Upper Leg, Thigh		Knee		Shoulder (incl Clavicle/blade)		Upper And Lower Arm	
Gender	Claim Count	Cost Ex GST	Claim Count	Cost Ex GST	Claim Count	Cost Ex GST	Claim Count	Cost Ex GST	Claim Count	Cost Ex GST	Claim Count	Cost Ex GST	Claim Count	Cost Ex GST	Claim Count	Cost Ex GST
Female	9420.00	8700554.00	2605.00	2009435.00	1465.00	1879472.00	18188.00	12460395.00	6238.00	3343524.00	21464.00	25856698.00	27283.00	28307045.00	339.00	577305.00
Male	10759.00	15342686.00	3631.00	4550417.00	1896.00	3709952.00	16355.00	16035710.00	10753.00	5652343.00	25849.00	50053256.00	37952.00	80448674.00	1276.00	5663766.00

Claim count and total costs for claims that received a payment in the 2014/15 financial year, with tendon and ligament injuries, broken down by client ethnicity and the injury site

	Ankle		Elbow		Finger/thumb		Hand/wrist		Hip, Upper Leg, Thigh		Knee		Shoulder (incl Clavicle/blade)		Upper And Lower Arm	
Ethnicity Prioritised	Claim Count	Cost Ex GST	Claim Count	Cost Ex GST	Claim Count	Cost Ex GST	Claim Count	Cost Ex GST	Claim Count	Cost Ex GST	Claim Count	Cost Ex GST	Claim Count	Cost Ex GST	Claim Count	Cost Ex GST
Asian	752	$1,008,854	562	$227,800	207	$226,857	3,301	$2,091,684	840	$343,246	3,086	$4,317,678	3,898	$3,786,597	48	$231,211
European	14,383	$15,953,679	4,358	$4,922,161	2,366	$3,907,172	23,485	$20,809,550	11,943	$6,822,898	31,875	$49,524,578	48,083	$86,490,704	1,232	$4,593,169
Maori	2,819	$4,320,841	594	$839,947	430	$865,548	3,826	$2,962,977	1,768	$788,636	5,996	$11,621,223	5,991	$10,644,316	212	$983,561
Other Ethnicity	1,139	$1,148,892	417	$417,574	185	$301,466	2,106	$1,609,922	1,181	$542,983	2,558	$4,313,208	3,951	$4,806,060	48	$196,102
Pacific Peoples	1,086	$1,610,975	305	$152,369	173	$288,381	1,825	$1,021,971	1,259	$498,104	3,798	$6,133,267	3,312	$3,028,042	75	$237,028

Claim count and total costs for claims that received a payment in the 2014/15 financial year, with tendon and ligament injuries, broken down by client's age at lodgement (in year bands) and the injury site

	Ankle		Elbow		Finger/thumb		Hand/wrist		Hip, Upper Leg, Thigh		Knee		Shoulder (incl Clavicle/blade)		Upper And Lower Arm	
Age at Lodgement	Claim Count	Cost Ex GST	Claim Count	Cost Ex GST	Claim Count	Cost Ex GST	Claim Count	Cost Ex GST	Claim Count	Cost Ex GST	Claim Count	Cost Ex GST	Claim Count	Cost Ex GST	Claim Count	Cost Ex GST
00-14 Years	2,092	$416,662	625	$118,609	337	$92,059	6,782	$1,557,856	1,787	$363,425	4,102	$1,984,352	1,711	$532,447	21	$4,048
15-19 Years	1,169	$556,902	258	$79,077	297	$228,340	3,639	$2,014,215	2,255	$543,406	4,689	$7,379,049	4,663	$2,634,052	48	$39,482
20-24 Years	974	$951,965	260	$170,764	326	$865,579	3,193	$3,142,877	1,851	$628,601	4,140	$10,904,431	4,843	$4,051,579	55	$185,858
25-29 Years	1,183	$1,874,387	284	$347,867	303	$666,703	3,021	$2,892,020	1,488	$738,418	3,456	$9,552,110	4,178	$4,145,662	58	$232,216
30-34 Years	1,405	$2,383,457	374	$667,498	253	$567,011	2,567	$3,125,550	1,238	$707,773	3,071	$8,211,551	3,846	$4,632,537	69	$337,079
35-39 Years	1,833	$2,872,766	554	$902,500	254	$454,597	2,312	$2,527,662	1,131	$595,563	3,152	$6,969,133	4,330	$6,950,868	89	$682,542
40-44 Years	2,355	$3,414,458	892	$1,164,341	288	$758,275	2,348	$3,318,505	1,388	$887,748	3,811	$8,152,906	5,792	$10,764,112	162	$1,020,110
45-49 Years	2,351	$3,058,230	1,020	$1,521,662	295	$605,148	2,188	$2,690,844	1,395	$1,216,915	4,101	$7,120,164	6,684	$15,541,251	172	$1,153,092
50-54 Years	2,101	$2,794,551	842	$796,931	256	$454,244	2,257	$2,713,230	1,258	$1,009,234	4,195	$5,529,407	7,252	$17,600,876	178	$897,863
55-59 Years	1,490	$1,950,418	517	$561,194	217	$361,808	1,864	$1,581,420	933	$785,054	3,720	$3,971,153	6,111	$17,318,097	173	$703,757
60-64 Years	1,155	$1,968,947	299	$116,985	190	$271,469	1,436	$1,467,016	804	$785,716	3,125	$2,919,913	5,140	$13,477,675	136	$582,303
65-69 Years	926	$882,647	157	$63,528	148	$168,150	1,076	$659,083	576	$373,807	2,476	$1,714,622	4,238	$6,485,957	136	$257,870
70-74 Years	556	$448,778	68	$15,558	87	$39,675	703	$407,548	400	$167,332	1,573	$800,325	2,867	$2,572,359	124	$77,376
75-79 Years	324	$250,172	46	$23,952	56	$32,282	526	$185,032	251	$116,802	907	$373,352	1,798	$1,127,570	88	$42,283
80-84 Years	174	$144,490	25	$6,470	33	$15,588	339	$138,247	150	$52,824	500	$218,512	1,081	$641,961	57	$15,206
85 Years and over	91	$74,409	15	$2,917	21	$8,497	292	$75,000	86	$23,248	295	$108,972	701	$278,716	49	$9,987

Claim count and total costs for claims that received a payment in the 2014/15 financial year, with tendon and ligament injuries, broken down by client gender and ethnicity

	Asian		European		Maori		Other Ethnicity		Pacific Peoples	
Gender	Claim Count	Cost Ex GST	Claim Count	Cost Ex GST	Claim Count	Cost Ex GST	Claim Count	Cost Ex GST	Claim Count	Cost Ex GST
Female	5,712	$4,444,169	63,548	$60,751,031	9,243	$10,087,395	4,748	$4,321,288	3,760	$3,528,118
Male	6,999	$7,809,028	74,307	$131,855,076	12,760	$23,633,402	6,269	$8,591,631	8,143	$9,639,673

Claim count and total costs for claims that received a payment in the 2014/15 financial year, with tendon and ligament injuries, broken down by injury site and ethnicity

	Asian		European		Maori		Other Ethnicity		Pacific Peoples	
Injury Site	Claim Count	Cost Ex GST	Claim Count	Cost Ex GST	Claim Count	Cost Ex GST	Claim Count	Cost Ex GST	Claim Count	Cost Ex GST
Ankle	750	$973,597	14,412	$15,873,018	2,856	$4,412,564	1,066	$1,135,510	1,098	$1,584,119
Elbow	570	$229,298	4,371	$4,801,349	608	$850,593	376	$464,148	310	$169,598
Finger/thumb	205	$226,781	2,377	$3,935,726	434	$867,771	173	$311,065	173	$288,323
Hand/wrist	3,311	$2,094,656	23,479	$20,740,745	3,907	$3,056,524	2,015	$1,553,452	1,829	$1,020,768
Hip, Upper Leg, Thigh	850	$349,943	11,952	$6,786,204	1,810	$793,864	1,116	$574,165	1,265	$497,671
Knee	3,072	$4,406,261	31,895	$49,394,475	6,068	$11,811,326	2,461	$4,166,222	3,825	$6,255,274
Shoulder (incl Clavicle/blade)	3,905	$3,741,539	48,136	$86,475,926	6,109	$10,957,505	3,760	$4,494,832	3,331	$3,125,558
Upper And Lower Arm	48	$231,121	1,233	$4,598,665	211	$970,649	50	$213,522	72	$226,480

Claim count and total costs for claims that received a payment in the 2014/15 financial year, with tendon and ligament injuries, broken down by client's age at lodgement (in year bands

	Asian		European		Maori		Other Ethnicity		Pacific Peoples	
Age at Lodgement	Claim Count	Cost Ex GST	Claim Count	Cost Ex GST	Claim Count	Cost Ex GST	Claim Count	Cost Ex GST	Claim Count	Cost Ex GST
00-14 Years	946	$266,954	11,757	$3,386,563	2,633	$839,234	912	$254,857	1,210	$322,089
15-19 Years	886	$551,266	9,997	$8,116,893	2,948	$2,503,349	961	$629,482	2,229	$1,684,615
20-24 Years	1,126	$1,005,168	9,287	$13,456,245	2,293	$3,380,330	1,031	$1,103,479	1,902	$1,949,368
25-29 Years	1,431	$1,379,153	8,129	$12,108,038	2,060	$3,860,559	980	$1,161,093	1,372	$1,934,414
30-34 Years	1,451	$1,714,790	7,562	$12,362,761	1,824	$3,395,284	920	$1,307,779	1,067	$1,711,428
35-39 Years	1,014	$1,193,857	8,843	$14,498,586	1,939	$3,659,705	917	$1,369,913	942	$1,247,403
40-44 Years	1,117	$1,334,137	12,135	$21,570,460	1,973	$3,773,521	991	$1,487,280	823	$1,367,636
45-49 Years	1,150	$1,450,081	13,434	$24,107,203	1,873	$4,324,333	998	$1,917,160	750	$1,122,917
50-54 Years	1,182	$1,367,547	14,014	$24,256,945	1,608	$4,053,866	972	$1,362,965	568	$811,845
55-59 Years	855	$783,149	11,810	$22,939,253	1,194	$2,160,391	754	$884,910	416	$535,512
60-64 Years	666	$638,098	10,068	$18,888,334	712	$1,024,766	560	$747,857	281	$294,885
65-69 Years	401	$349,422	8,292	$9,293,109	447	$500,555	441	$380,758	152	$81,820
70-74 Years	217	$89,000	5,497	$4,062,771	280	$163,990	280	$170,301	104	$42,887
75-79 Years	175	$83,691	3,477	$1,908,242	140	$53,057	146	$74,939	58	$31,517
80-84 Years	67	$34,237	2,123	$1,129,787	57	$13,462	90	$28,095	22	$27,718
85 Years and over	27	$12,648	1,430	$520,917	22	$14,396	64	$32,048	7	$1,738

Claim count and total costs for claims that received a payment in the 2015/16 financial year, with tendon and ligament injuries, broken down by client gender and the injury site

	Ankle		Elbow		Finger/thumb		Hand/wrist		Hip, Upper Leg, Thigh		Knee		Shoulder (incl Clavicle/blade)		Upper And Lower Arm	
Gender	Claim Count	Cost Ex GST	Claim Count	Cost Ex GST	Claim Count	Cost Ex GST	Claim Count	Cost Ex GST	Claim Count	Cost Ex GST	Claim Count	Cost Ex GST	Claim Count	Cost Ex GST	Claim Count	Cost Ex GST
Female	9549.00	9028201.00	2333.00	2519506.00	1473.00	1992370.00	19630.00	13958972.00	6482.00	3516460.00	22309.00	27757732.00	27447.00	29091371.00	346.00	735153.00
Male	10708.00	16589824.00	3411.00	4608374.00	1896.00	4575412.00	17035.00	17580359.00	11164.00	6641020.00	26093.00	53552792.00	37436.00	84507943.00	1268.00	6679443.00
	20,257	25,618,025	5,744	7,127,880	3,369	6,567,782	36,665	31,539,331	17,646	10,157,480	48,402	81,310,524	64,883	113,599,314	1,614	7,414,596

Claim count and total costs for claims that received a payment in the 2015/16 financial year, with tendon and ligament injuries, broken down by client ethnicity and the injury site

	Ankle		Elbow		Finger/thumb		Hand/wrist		Hip, Upper Leg, Thigh		Knee		Shoulder (incl Clavicle/blade)		Upper And Lower Arm	
Ethnicity Prioritised	Claim Count	Cost Ex GST	Claim Count	Cost Ex GST	Claim Count	Cost Ex GST	Claim Count	Cost Ex GST	Claim Count	Cost Ex GST	Claim Count	Cost Ex GST	Claim Count	Cost Ex GST	Claim Count	Cost Ex GST
Asian	783	$958,664	566	$316,306	208	$267,631	3,774	$2,216,267	968	$560,368	3,224	$4,236,600	4,192	$4,328,419	35	$170,338
European	14,295	$16,977,503	3,911	$5,019,403	2,372	$4,415,779	24,649	$22,276,038	12,380	$7,672,722	32,746	$53,621,549	47,440	$89,048,749	1,243	$5,644,672
Maori	2,915	$4,920,242	580	$938,967	428	$1,089,567	4,094	$3,478,251	1,847	$954,570	6,132	$12,063,181	6,006	$11,247,866	185	$1,118,384
Other Ethnicity	1,180	$1,165,468	386	$708,901	205	$482,011	2,239	$2,111,690	1,221	$522,499	2,735	$4,783,321	3,884	$4,849,412	63	$212,310
Pacific Peoples	1,084	$1,596,148	301	$144,303	156	$312,794	1,909	$1,457,084	1,230	$447,321	3,565	$6,605,872	3,361	$4,124,870	88	$268,893

Claim count and total costs for claims that received a payment in the 2015/16 financial year, with tendon and ligament injuries, broken down by client's age at lodgement (in year bands) and the injury site

	Ankle		Elbow		Finger/thumb		Hand/wrist		Hip, Upper Leg, Thigh		Knee		Shoulder (incl Clavicle/blade)		Upper And Lower Arm	
Age at Lodgement	Claim Count	Cost Ex GST	Claim Count	Cost Ex GST	Claim Count	Cost Ex GST	Claim Count	Cost Ex GST	Claim Count	Cost Ex GST	Claim Count	Cost Ex GST	Claim Count	Cost Ex GST	Claim Count	Cost Ex GST
00-14 Years	2,066	$387,022	561	$129,849	326	$100,005	7,312	$1,780,430	1,667	$338,885	4,319	$2,257,772	1,664	$531,603	28	$10,544
15-19 Years	1,109	$480,854	245	$55,429	260	$344,254	3,631	$2,043,744	2,233	$540,602	4,530	$7,110,626	4,388	$2,634,222	35	$16,662
20-24 Years	953	$1,181,870	254	$140,937	303	$677,964	3,318	$3,332,807	1,783	$653,101	3,997	$10,833,484	4,462	$4,975,525	62	$260,719
25-29 Years	1,227	$1,900,941	256	$191,666	340	$620,282	3,218	$3,474,030	1,615	$735,258	3,445	$9,299,638	4,329	$4,786,566	49	$393,472
30-34 Years	1,401	$2,325,886	390	$705,494	240	$703,351	2,889	$3,473,671	1,245	$644,324	3,145	$8,257,613	3,899	$4,934,549	67	$459,884
35-39 Years	1,800	$2,984,427	475	$826,952	235	$781,036	2,436	$2,746,585	1,156	$612,659	3,143	$7,538,171	4,345	$7,617,465	89	$844,621
40-44 Years	2,261	$3,887,370	874	$1,626,208	266	$915,724	2,461	$3,441,368	1,395	$1,060,924	3,766	$8,397,518	5,650	$11,140,228	151	$1,060,938
45-49 Years	2,377	$2,871,857	851	$1,425,402	301	$646,880	2,353	$3,547,985	1,494	$1,231,547	4,195	$8,611,505	6,804	$16,149,696	178	$1,130,706
50-54 Years	2,152	$3,463,291	768	$1,145,658	307	$717,312	2,351	$2,778,418	1,406	$1,441,142	4,250	$6,883,325	7,221	$18,013,898	181	$1,373,327
55-59 Years	1,500	$1,997,820	489	$596,440	235	$411,774	2,030	$2,006,040	1,108	$952,653	3,979	$5,068,363	6,183	$18,046,624	153	$742,933
60-64 Years	1,259	$2,281,931	285	$145,819	196	$367,769	1,501	$1,542,201	802	$811,420	3,413	$3,434,113	5,047	$13,106,231	160	$610,900
65-69 Years	1,001	$990,125	152	$100,995	164	$151,643	1,150	$583,955	717	$608,605	2,677	$1,913,199	4,353	$6,619,955	149	$351,014
70-74 Years	583	$507,714	72	$14,587	89	$57,308	806	$347,400	477	$280,564	1,689	$841,969	2,887	$2,946,264	109	$97,423
75-79 Years	346	$223,909	39	$11,991	49	$29,741	572	$223,975	304	$141,081	1,022	$518,233	1,862	$1,156,875	86	$30,160
80-84 Years	153	$92,684	18	$6,526	33	$36,634	330	$131,109	146	$76,411	516	$222,149	1,060	$631,324	55	$17,881
85 Years and over	69	$40,324	15	$3,928	25	$6,103	307	$85,613	98	$28,306	316	$122,845	729	$308,291	62	$13,413

Claim count and total costs for claims that received a payment in the 2015/16 financial year, with tendon and ligament injuries, broken down by client gender and ethnicity

	Asian		European		Maori		Other Ethnicity		Pacific Peoples	
Gender	Claim Count	Cost Ex GST	Claim Count	Cost Ex GST	Claim Count	Cost Ex GST	Claim Count	Cost Ex GST	Claim Count	Cost Ex GST
Female	6,353	$4,861,398	64,942	$64,159,053	9,603	$11,494,549	4,893	$4,431,943	3,789	$3,680,915
Male	7,423	$8,055,207	74,192	$139,900,951	13,019	$25,362,536	6,439	$9,994,478	7,962	$11,582,709

Claim count and total costs for claims that received a payment in the 2015/16 financial year, with tendon and ligament injuries, broken down by injury site and ethnicity

	Asian		European		Maori		Other Ethnicity		Pacific Peoples	
Injury Site	Claim Count	Cost Ex GST	Claim Count	Cost Ex GST	Claim Count	Cost Ex GST	Claim Count	Cost Ex GST	Claim Count	Cost Ex GST
Ankle	777	$878,918	14,336	$16,835,441	2,953	$5,059,028	1,099	$1,221,195	1,100	$1,572,520
Elbow	576	$322,074	3,915	$4,981,233	600	$975,498	355	$649,633	299	$143,988
Finger/thumb	210	$257,731	2,384	$4,442,597	429	$1,117,642	192	$425,162	158	$324,189
Hand/wrist	3,780	$2,228,537	24,647	$22,185,395	4,183	$3,575,986	2,123	$1,984,263	1,931	$1,495,602
Hip, Upper Leg, Thigh	968	$558,199	12,406	$7,562,783	1,881	$962,362	1,159	$625,925	1,232	$446,799
Knee	3,241	$4,230,735	32,717	$53,360,444	6,251	$12,408,624	2,646	$4,707,977	3,558	$6,736,771
Shoulder (incl Clavicle/blade)	4,188	$4,269,788	47,480	$89,068,336	6,138	$11,624,924	3,703	$4,608,457	3,385	$4,261,028
Upper And Lower Arm	36	$170,621	1,249	$5,623,774	187	$1,133,021	55	$203,810	88	$282,727

Claim count and total costs for claims that received a payment in the 2015/16 financial year, with tendon and ligament injuries, broken down by client's age at lodgement (in year bands

	Asian		European		Maori		Other Ethnicity		Pacific Peoples	
Age at Lodgement	Claim Count	Cost Ex GST	Claim Count	Cost Ex GST	Claim Count	Cost Ex GST	Claim Count	Cost Ex GST	Claim Count	Cost Ex GST
00-14 Years	1,056	$338,063	12,173	$3,742,026	2,730	$751,329	883	$279,649	1,102	$425,715
15-19 Years	884	$409,274	9,557	$8,178,659	2,855	$2,515,052	935	$522,888	2,206	$1,663,001
20-24 Years	1,179	$850,205	8,871	$14,163,218	2,335	$3,836,618	1,025	$1,134,621	1,721	$2,077,168
25-29 Years	1,526	$1,479,770	8,220	$12,154,567	2,181	$4,085,959	1,116	$1,467,558	1,443	$2,294,750
30-34 Years	1,577	$1,736,330	7,690	$12,724,624	1,931	$3,865,347	1,013	$1,277,432	1,067	$1,736,826
35-39 Years	1,187	$1,252,040	8,777	$15,613,557	1,859	$3,882,467	912	$1,573,165	943	$1,629,696
40-44 Years	1,180	$1,446,733	11,848	$22,666,786	2,007	$3,931,349	990	$1,945,649	803	$1,521,894
45-49 Years	1,177	$1,577,135	13,539	$25,406,647	1,967	$4,974,299	1,127	$1,880,600	745	$1,812,009
50-54 Years	1,253	$1,450,130	14,102	$27,850,093	1,691	$3,947,236	977	$1,776,994	620	$842,950
55-59 Years	945	$959,243	12,359	$24,686,699	1,245	$2,750,224	739	$867,513	393	$640,133
60-64 Years	783	$771,421	10,212	$18,940,402	777	$1,408,288	573	$899,553	321	$335,628
65-69 Years	503	$347,130	8,687	$9,720,491	527	$592,127	461	$499,276	187	$161,157
70-74 Years	259	$159,264	5,788	$4,524,239	272	$201,897	283	$160,901	110	$46,929
75-79 Years	180	$105,193	3,702	$2,030,014	166	$85,946	170	$91,116	61	$23,340
80-84 Years	61	$24,089	2,107	$1,084,304	54	$21,254	69	$34,298	20	$50,773
85 Years and over	26	$10,587	1,502	$573,679	25	$7,693	59	$15,207	9	$1,657
