# Supplementary material for: Epidemiology of tendon and ligament injuries in Aotearoa/New Zealand between 2010 and 2016
Source: Inj Epidemiol. 2020 Feb 10;7:5. doi: 10.1186/s40621-020-0231-x (PMC7008565; doi:10.1186/s40621-020-0231-x)
Supplement: Supplementary file 2 — Additional file 2. Copy of Aotearoa/New Zealand population by prioritised ethnicity and age groups as of the 2013 census, received from Statistics NZ [file 40621_2020_231_MOESM2_ESM.pdf]

New Zealand

Resident Population by Prioritised Ethnicity and Age Groups at 30 June 2013

Prioritised	Age Group Population																		
Ethnic Group	0-4	5-9	10-14	15-19	20-24	25-29	30-34	35-39	40-44	45=49	50-54	55-59	60-64	65-69	70-74	75-79	80-84	85 and Over	Total
Maori	84,040	77,160	71,780	68,880	58,880	44,670	39,770	40,260	42,900	39,520	37,750	28,570	21,620	15,030	10,210	6,140	3,360	1,730	692,300
Pacific	30,740	28,340	28,590	28,260	25,800	20,450	18,910	18,230	18,600	17,450	14,420	11,090	9,010	6,430	4,370	2,700	1,520	910	285,800
Asian	37,200	30,850	30,360	39,370	53,970	56,370	53,160	39,220	38,150	35,200	31,220	25,820	18,870	12,450	8,590	5,790	2,940	1,620	521,100
European	152,050	156,060	159,440	169,700	168,040	147,120	149,870	169,690	207,090	210,830	221,740	199,310	186,100	167,670	128,300	93,370	73,600	69,960	2,829,900
Other	7,900	7,660	6,590	6,280	8,230	8,870	9,380	8,970	9,830	9,000	8,550	6,970	5,410	4,180	2,570	1,340	700	510	112,900
Total	311,930	300,070	296,770	312,480	314,920	277,490	271,090	276,360	316,570	311,990	313,670	271,760	241,010	205,760	154,040	109,340	82,120	74,730	4,442,100

Note: Owing to rounding, individual figures do not always sum to the stated toals.

Source: Stats NZ
