# Supplementary material for: Epidemiology of tendon and ligament injuries in Aotearoa/New Zealand between 2010 and 2016
Source: Inj Epidemiol. 2020 Feb 10;7:5. doi: 10.1186/s40621-020-0231-x (PMC7008565; doi:10.1186/s40621-020-0231-x)
Supplement: Supplementary file 3 — Additional file 3. List of all tendon/ligament injury diagnoses included in the study [file 40621_2020_231_MOESM3_ESM.pdf]

List of injuries identified when using key words as outlined by customer

Final Diagnosis
Abscess of tendon sheath, ankle and foot
Abscess of tendon sheath, hand
Abscess of tendon sheath, lower leg
Abscess of tendon sheath, pelvic region and thigh
Achilles Bursitis or Tendinitis
Achilles tendinitis
Achilles tenosynovitis
Adductor tendinitis
Ankle or tarsus enthesopathy NOS
Biceps tendinitis
Biceps tendon rupture
Bicipital tendinitis
Bicipital tenosynovitis
Calcific tendinitis of shoulder
Calcific tendinitis, other
Calcific tendinitis, upper arm
Calcifying Tendinitis of Shoulder
Calcifying tendinitis of the shoulder
Complete division extensor tendon hand
Complete division extensor tendon wrist
Complete division flexor tendon hand
Complete division flexor tendon wrist
Complete division, biceps tendon
Complete division, both flexor tendons
Complete division, extensor digitorum tendon
Complete division, extensor pollicis longus tendon
Complete division, flexor digitorum profundus tendon
Complete division, flexor digitorum superficialis tendon
Complete division, flexor pollicis longus tendon
Complete division, hamstring tendon
Complete division, long head of biceps tendon
Complete division, patellar tendon
Complete division, quadriceps tendon
Complete division, tendocalcaneus (Achilles tendon)
Complete division, triceps tendon
Complete tear Iliofemoral ligament
Complete tear dorsal intercarpal ligament
Complete tear dorsal radio-carpal ligament
Complete tear finger D.I.P.J. radial collateral ligament
Complete tear finger D.I.P.J. ulnar collateral ligament
Complete tear finger M.C.P.J. radial collateral ligament
Complete tear finger M.C.P.J. ulnar collateral ligament
Complete tear finger P.I.P.J. radial collateral ligament
Complete tear finger P.I.P.J. ulnar collateral ligament
Complete tear hip ligament NOS
Complete tear ligament finger
Complete tear ligament finger NOS
Complete tear ligament thumb
Complete tear ligament thumb NOS
Complete tear luno-triquetral ligament
Complete tear other hip ligament

Complete tear thumb I.P.J. radial collateral ligament
Complete tear thumb I.P.J. ulnar collateral ligament
Complete tear thumb M.C.P.J. radial collateral ligament
Complete tear thumb M.C.P.J. ulnar collateral ligament
Complete tear ulnar collateral ligament
Complete tear volar intercarpal ligament (V ligament)
Complete tear volar radio-carpal ligament non-specific
Complete tear volar radio-carpal ligament superficial
Complete tear wrist ligament
Complete tear wrist ligament NOS
Complete tear, acromio-clavicular ligament
Complete tear, ankle ligament
Complete tear, ankle ligament NOS
Complete tear, ankle or foot ligament
Complete tear, ankle or foot ligament NOS
Complete tear, ankle, lateral ligament
Complete tear, ankle, medial ligament
Complete tear, coraco-clavicular ligament
Complete tear, elbow joint, lateral collateral ligament
Complete tear, elbow joint, medial collateral ligament
Complete tear, foot ligament
Complete tear, hip ligament
Complete tear, knee ligament
Complete tear, knee ligament NOS
Complete tear, knee, anterior cruciate ligament
Complete tear, knee, lateral collateral ligament
Complete tear, knee, medial collateral ligament
Complete tear, knee, posterior cruciate ligament
Complete tear, metatarsophalangeal ligament
Complete tear, other knee ligament
Complete tear, sternoclavicular ligament
Dislocation of tendon, wrist or hand
Disorders of Bursae and Tendons in Shoulder Region, Unspecif
Disorders of Bursae and Tendons in Shoulder Region, Unspecified; Rotator Cuff Syndrome NOS; Supraspinatus Syndrome NOS
Elbow enthesopathy NOS
Elbow enthesopathy unspecified
Enthesopathy of the ankle unspecified
Enthesopathy of the elbow region
Enthesopathy of the hip region
Enthesopathy of the knee
Enthesopathy of the wrist and carpus
Epicondylitis, Lateral
Epicondylitis, Medial
Extensor tenosynovitis of finger
Extensor tenosynovitis of thumb
Extensor tenosynovitis of wrist
Flexor Tendon of Hand, Delayed Suture
Flexor Tendon of Hand, Other Suture
Flexor tenosynovitis of finger
Flexor tenosynovitis of thumb
Flexor tenosynovitis of wrist
Full thickness rotator cuff tear
Ganglion and Cyst of Synovium, Tendon and Bursa
Ganglion and cyst of synovium, tendon and bursa
Ganglion of flexor tendon sheath of finger
Gluteal tendinitis

Inj muscle & tendon long flex musc toe at ankle & foot level
Inj of other & unspecif musc & tendons at forearm level
Inj of unspecified muscle & tendon at wrist & hand level
Inj/extens or abduct muscle+tendon/thumb at forearm level
Inj/extensor muscle+tendon/oth finger(s) at forearm level
Inj/flex muscle and tendon of other finger(s)/forearm level
Inj/muscle + tendon/long exten musc/toe at ankle + ft level
Injury of Achilles tendon
Injury of adductor muscle and tendon of thigh
Injury of extensor muscle and tendon of other finger at wrist and hand level
Injury of extensor muscle and tendon of other finger(s) at forearm level
Injury of extensor muscle and tendon of thumb at wrist and hand level
Injury of extensor or abductor muscles and tendons of thumb at forearm level
Injury of flexor muscle and tendon of other finger at wrist and hand level
Injury of flexor muscle and tendon of other finger(s) at forearm level
Injury of flexor muscle and tendon of thumb at forearm level
Injury of intrinsic muscle and tendon of other finger at wrist and hand level
Injury of intrinsic muscle and tendon of thumb at wrist and hand level
Injury of long flexor muscle and tendon of other finger(s) at forearm level
Injury of long flexor muscle and tendon of thumb at forearm level
Injury of long flexor muscle and tendon of thumb at wrist and hand level
Injury of multiple extensor muscles and tendons at wrist and hand level
Injury of multiple flexor muscles and tendons at wrist and hand level
Injury of multiple muscles and tendons at forearm level
Injury of multiple muscles and tendons at hip + thigh level
Injury of multiple muscles and tendons at hip and thigh level
Injury of multiple muscles and tendons at lower leg level
Injury of multiple muscles and tendons at shoulder and upper arm level
Injury of muscle and tendon at ankle and foot level
Injury of muscle and tendon at forearm level
Injury of muscle and tendon at hip and thigh level
Injury of muscle and tendon at neck level
Injury of muscle and tendon of hip
Injury of muscle and tendon of long head of biceps
Injury of muscle and tendon of other parts of biceps
Injury of muscle and tendon of the posterior muscle group at thigh level
Injury of muscle and tendon of triceps
Injury of muscle(s) and tendon(s) of anterior muscle group at lower leg level
Injury of muscle(s) and tendon(s) of peroneal muscle group at lower leg level
Injury of other and unspecified muscles and tendons at forearm level
Injury of other and unspecified muscles and tendons at thigh level
Injury of other extensor muscle and tendon at forearm level
Injury of other flexor muscle and tendon at forearm level
Injury of other muscle(s) and tendon(s) of posterior muscle group at lower leg level
Injury of other muscles and tendons at lower leg level
Injury of other muscles and tendons at shoulder and upper arm level
Injury of other muscles and tendons at wrist and hand level
Injury of quadriceps muscle and tendon
Injury of tendon of the rotator cuff of shoulder
Injury of unspecified muscle & tendon of ankle and foot
Injury of unspecified muscle and tendon at ankle and foot level
Injury of unspecified muscle and tendon at lower leg level
Injury of unspecified muscle and tendon at lower leg levl
Injury of unspecified muscle and tendon at shoulder and upper arm level
Injury of unspecified muscle and tendon at wrist and hand level
Injury of unspecified muscle and tendon of lower limb, level unspecified

Loose body, unspecified ligament or unspecified meniscus
Mallet finger with closed tendon injury
Mallet finger with open tendon injury
Mallet thumb with closed tendon injury
Mallet thumb with open tendon injury
Medial epicondylitis
Medial epicondylitis of the elbow
Multiple open wounds of upper arm with tendon involvement
Multiple/unspec open wound upper limb with tendon involved
Old anterior cruciate ligament disruption
Old complete tear anterior cruciate ligament
Old complete tear lateral collateral ligament
Old complete tear medial collateral ligament
Old complete tear posterior cruciate ligament
Old medial collateral ligament disruption
Old partial tear anterior cruciate ligament
Old posterior cruciate ligament disruption
Open Wound of Hand except Finger(s), with Tendon Involvement
Open Wound of Wrist, with Tendon Involvement
Open Wound, Elbow, Forearm, and Wrist, with Tendon Involvement, Forearm
Open Wound, Finger(s), with Tendon Involvement
Open Wound, Hand, Except Finger(s) Alone, with Tendon Complication
Open division acromioclavicular ligament
Open division ankle ligament
Open division ankle ligament NOS
Open division ankle or foot ligament NOS
Open division ankle, lateral ligament
Open division ankle, medial ligament
Open division anterior cruciate ligament knee
Open division calcaneofibular ligament
Open division distal tibiofibular ligament
Open division elbow ligament
Open division elbow ligament NOS
Open division elbow, lateral collateral ligament
Open division elbow, medial collateral ligament
Open division finger DIPJ, radial collateral ligament
Open division finger DIPJ, ulnar collateral ligament
Open division finger MCPJ, radial collateral ligament
Open division finger MCPJ, ulnar collateral ligament
Open division finger PIPJ, radial collateral ligament
Open division finger PIPJ, ulnar collateral ligament
Open division finger ligament
Open division finger ligament NOS
Open division hip ligament
Open division iliofemoral ligament
Open division knee ligament NOS
Open division lateral collateral ligament knee
Open division ligament ankle or foot
Open division ligament knee
Open division medial collateral ligament knee
Open division other elbow ligament
Open division other knee ligament
Open division posterior cruciate ligament knee
Open division radiohumeral ligament
Open division shoulder ligament
Open division shoulder ligament NOS

Open division ulnohumeral ligament
Open division wrist ligament
Open division wrist ligament NOS
Open division wrist ligament, single
Open division wrist ligaments, multiple
Open division wrist or hand ligament
Open division wrist or hand ligament NOS
Open division, superior tibiofibular ligament
Open wound of ankle with tendon involvement
Open wound of elbow with tendon involvement
Open wound of finger or thumb with tendon involvement
Open wound of finger with tendon injury
Open wound of forearm with tendon involvement
Open wound of hand with tendon involvement
Open wound of hip and thigh with tendon involvement
Open wound of hip with tendon involvement
Open wound of knee with tendon involvement
Open wound of knee/leg/ankle with tendon involvement, NOS
Open wound of lower arm with tendon involvement
Open wound of lower arm with tendon involvement, NOS
Open wound of lower leg with tendon involvement
Open wound of shoulder region with tendon involvement
Open wound of shoulder/upper limb with tendon involvement
Open wound of thigh with tendon involvement
Open wound of upper arm with tendon involvement
Open wound of wrist with tendon involvement
Open wound shoulder/upper arm with tendon involvement, NOS
Other Tendon of Hand, Delayed Suture
Other Tenosynovitis of Hand and Wrist
Other contracture of tendon (sheath), hand
Other internal derangements of knee, unspecified ligament or unspecified meniscus
Other internal derangements of lateral collateral ligament or anterior horn of lateral meniscus
Other internal derangements of medial collateral ligament or other and unspecified medial meniscus
Other internal derangements of posterior cruciate ligament or posterior horn of medial meniscus
Other muscle, ligament or fascia disorder NOS
Other old knee ligament disruption
Other synovitis and tenosynovitis
Other synovitis and tenosynovitis, forearm
Other synovitis and tenosynovitis, hand
Other synovitis and tenosynovitis, lower leg
Other synovitis and tenosynovitis, multiple sites
Other synovitis and tenosynovitis, shoulder region
Other synovitis and tenosynovitis, upper arm
Other tenosynovitis of hand or wrist
Partial division both flexor tendons
Partial division extensor tendon hand
Partial division extensor tendon wrist
Partial division flexor tendon hand
Partial division flexor tendon wrist
Partial division, biceps tendon
Partial division, extensor digitorum tendon
Partial division, extensor pollicis longus tendon
Partial division, flexor digitorum profundus tendon
Partial division, flexor digitorum superficialis tendon
Partial division, flexor pollicis longus tendon
Partial division, hamstring tendon

Partial tear, ankle, medial ligament
Partial tear, knee, anterior cruciate ligament
Partial tear, knee, lateral collateral ligament
Partial tear, knee, medial collateral ligament
Partial tear, knee, posterior cruciate ligament
Partial thickness rotator cuff tear
Patellar tendinitis
Peroneal tendinitis
Plastic repair of rotator cuff of shoulder
Psoas tendinitis
Quadriceps tendon rupture
Radial Styloid Tenosynovitis
Radial Styloid Tenosynovitis; de Quervain's Disease
Radial styloid tenosynovitis
Radial styloid tenosynovitis [de Quervain]
Repair, Anterior Cruciate Ligament
Rotator cuff complete rupture
Rotator cuff shoulder syndrome and allied disorders
Rotator cuff sprain
Rotator cuff syndrome
Rotator cuff syndrome NOS
Rotator cuff syndrome, unspecified
Rupture achilles tendon
Rupture biceps tendon
Rupture extensor digitorum tendon
Rupture flexor digitorum profundus tendon
Rupture flexor digitorum superficialis tendon
Rupture hamstring tendon
Rupture infraspinatus tendon
Rupture long head biceps tendon
Rupture of Tendon, Nontraumatic, Achilles Tendon
Rupture of Tendon, Nontraumatic, Biceps Tendon (Long Head)
Rupture of Tendon, Nontraumatic, Complete Rupture of Rotator Cuff
Rupture of Tendon, Nontraumatic, Extensor Tendons of Hand and Wrist
Rupture of Tendon, Nontraumatic, Flexor Tendons of Hand and Wrist
Rupture of anterior cruciate ligament
Rupture of lateral collateral ligament
Rupture of ligaments at ankle and foot level
Rupture of medial collateral ligament
Rupture of posterior cruciate ligament
Rupture patellar tendon
Rupture quadriceps tendon
Rupture subscapularis tendon
Rupture supraspinatus tendon
Rupture tendon forearm or wrist
Rupture tendon hand or wrist NOS
Rupture tendon of finger
Rupture tendon of finger NOS
Rupture tendon of thumb
Rupture tendon thigh
Rupture tendon thumb NOS
Rupture tendon upper arm
Rupture tendon upper arm NOS
Rupture triceps tendon
Rupture upper leg tendon NOS
Ruptured Biceps Tendon (Traumatic and Non-Traumatic)

Spontaneous rupture of extensor tendons, shoulder region
Spontaneous rupture of extensor tendons, upper arm
Spontaneous rupture of flexor tendons, hand
Spontaneous rupture of flexor tendons, lower leg
Spontaneous rupture of other tendons, lower leg
Spontaneous rupture of other tendons, pelvic region and thigh
Spontaneous rupture of other tendons, shoulder region
Spontaneous rupture of unspecified tendon, ankle and foot
Spontaneous rupture of unspecified tendon, lower leg
Spontaneous rupture of unspecified tendon, other site
Spontaneous rupture of unspecified tendon, pelvic region and thigh
Spontaneous rupture of unspecified tendon, shoulder region
Spontaneous rupture of unspecified tendon, site unspecified
Spontaneous rupture of unspecified tendon, upper arm
Sprain and strain iliofemoral (ligament)
Sprain and strain of anterior cruciate ligament
Sprain and strain of calcaneofibular (ligament)
Sprain and strain of deltoid (ligament), ankle
Sprain and strain of iliofemoral (ligament)
Sprain and strain of lateral collateral ligament
Sprain and strain of medial collateral ligament
Sprain and strain of posterior cruciate ligament
Sprain and strain of radial collateral ligament
Sprain and strain of radiocarpal (joint) (ligament)
Sprain and strain of tibiofibular (ligament)
Sprain and strain of ulna collateral ligament
Sprain and strain of unspecified collateral ligament
Sprain and strain of unspecified cruciate ligament
Sprain dorsal intercarpal ligament
Sprain dorsal radio-carpal ligament
Sprain finger D.I.P.J. radial collateral ligament
Sprain finger D.I.P.J. ulnar collateral ligament
Sprain finger M.C.P.J. radial collateral ligament
Sprain finger M.C.P.J. ulnar collateral ligament
Sprain finger P.I.P.J. radial collateral ligament
Sprain finger P.I.P.J. ulnar collateral ligament
Sprain luno-triquetral ligament
Sprain of cruciate ligament of knee
Sprain of medial collateral ligament of knee
Sprain of superior tibiofibular ligament
Sprain or partial tear, knee, lateral collateral ligament
Sprain proximal radiocarpal ligament non-specific
Sprain radial collateral ligament
Sprain radio-lunate ligament
Sprain radio-scapho-capitate ligament
Sprain radio-scapho-lunate ligament
Sprain scapho-lunate ligament
Sprain scapho-trapezium ligament
Sprain short intrinsic ligament non-specific
Sprain tendon of finger
Sprain tendon of thumb
Sprain tendon wrist or hand
Sprain thumb I.P.J. radial collateral ligament
Sprain thumb I.P.J. ulnar collateral ligament
Sprain thumb M.C.P.J. radial collateral ligament
Sprain thumb M.C.P.J. ulnar collateral ligament

Sprain, acromio-clavicular ligament
Sprain, biceps tendon
Sprain, coraco-clavicular ligament
Sprain, elbow joint, lateral collateral ligament
Sprain, elbow joint, medial collateral ligament
Sprain, elbow joint, radial collateral ligament
Sprain, elbow joint, ulnar collateral ligament
Sprain, extensor digitorum tendon
Sprain, extensor pollicis longus tendon
Sprain, flexor digitorum profundus tendon
Sprain, flexor digitorum superficialis tendon
Sprain, flexor pollicis longus tendon
Sprain, hamstring tendon
Sprain, infraspinatus tendon
Sprain, knee joint, lateral collateral ligament
Sprain, long head of biceps tendon
Sprain, patellar tendon
Sprain, plantaris tendon
Sprain, quadriceps tendon
Sprain, subscapularis tendon
Sprain, supraspinatus tendon
Sprain, tendocalcaneus (Achilles tendon)
Sprain, triceps tendon
Sprains and Strains of Ankle, Deltoid Ligament
Sprains and Strains of Ankle, Other (Achilles' Tendon)
Sprains and Strains of Ankle, Tibiofibular Ligament
Sprains and Strains of Biceps Tendon
Sprains and Strains of Cruciate Ligament of Knee
Sprains and Strains of Iliofemoral Ligament (Groin Strain)
Sprains and Strains of Knee and Leg, Cruciate Ligament of Knee
Sprains and Strains of Knee and Leg, Medial Collateral Ligament of Knee
Sprains and Strains of Medial Collateral Ligament of Knee
Sprains and Strains of Shoulder and Upper Arm, Acromioclavicular Joint (Ligament)
Sprains and Strains of Shoulder and Upper Arm, Coracohumeral (Ligament)
Sprains and Strains of Shoulder and Upper Arm, Supraspinatus (Muscle) (Tendon)
Sprains and Strains of Ulnar Collateral Ligament
Sprains and Strains, Ankle, Calcaneofibular Ligament
Sprains and Strains, Ankle, Deltoid (Ligament) Ankle; Internal Collateral (Ligament) Ankle
Sprains and Strains, Ankle, Tibiofibular Ligament, Distal
Subluxation of tendon, wrist or hand
Supraspinatus tendinitis
Synovitis and tenosynovitis
Tendon Release
Tendon Sheath of Hand, Suture
Tendon injury - hand
Tendon injury to hand NOS
Tendon sheath giant cell tumor
Tendonitis bicepital
Tenosynovitis and Synovitis
Tenosynovitis of ankle
Tibialis posterior tendinitis
Torn achilles tendon
Traumatic rupture of biceps tendon
Traumatic rupture of ligament of finger at metacarpophalangeal and interphalangeal joint(s)
Traumatic rupture of ligament of wrist and carpus
Traumatic rupture of radial collateral ligament

Unspecified internal derangement of medial collateral ligament or other and unspecified medial meniscus
Wrist or carpus enthesopathy NOS
[SO]Annular ligament
[SO]Anterior cruciate ligament
[SO]Ligament of ankle
[SO]Ligament of knee
[X]Inj extensor musc/tendon of oth finger(s) at forearm lev
[X]Inj intrins musc & tendon oth finger at wrist & hand lev
[X]Inj of oth muscles & tendons at should & upper arm level
[X]Inj of other & unspecif musc & tendons at forearm level
[X]Inj of unspecif musc & tendon at should & upper arm lev
[X]Inj of unspecified muscle & tendon at wrist & hand level
[X]Injury of oth extensor muscle & tendon at forearm level
[X]Injury of other & unspecif musc & tendons at thigh level
[X]Injury of other flexor muscle & tendon at forearm level
[X]Injury of other muscles & tendons at ankle & foot level
[X]Injury of other muscles & tendons at wrist & hand level
[X]Injury of unspec muscle & tendon of upr limb level unspec
[X]Injury of unspecified muscle & tendon of ankle and foot
[X]Other spontaneous disruption of ligament(s) of knee
